# Huntingtin Co-Isolates with Small Extracellular Vesicles from Blood Plasma of TgHD and KI-HD Pig Models of Huntington’s Disease and Human Blood Plasma

**DOI:** 10.3390/ijms23105598

**Published:** 2022-05-17

**Authors:** Hanadi Ananbeh, Jaromir Novak, Stefan Juhas, Jana Juhasova, Jiri Klempir, Kristyna Doleckova, Irena Rysankova, Karolina Turnovcova, Jaroslav Hanus, Hana Hansikova, Petr Vodicka, Helena Kupcova Skalnikova

**Affiliations:** 1 Institute of Animal Physiology and Genetics of the Czech Academy of Sciences, Rumburska 89, 27721 Libechov, Czech Republic; ananbeh@iapg.cas.cz (H.A.); novak@iapg.cas.cz (J.N.); juhas@iapg.cas.cz (S.J.); juhasova@iapg.cas.cz (J.J.); vodicka@iapg.cas.cz (P.V.); 2 Department of Cell Biology, Faculty of Science, Charles University, Vinicna 7, 12800 Prague, Czech Republic; 3 Department of Neurology and Center of Clinical Neuroscience, 1st Faculty of Medicine, Charles University and General University Hospital in Prague, Katerinska 30, 12821 Prague, Czech Republic; jiri.klempir@vfn.cz (J.K.); kristyna.doleckova@vfn.cz (K.D.); irena.rysankova@vfn.cz (I.R.); 4 Institute of Experimental Medicine of the Czech Academy of Sciences, Videnska 1083, 14220 Prague, Czech Republic; karolina.turnovcova@iem.cas.cz; 5 Department of Chemical Engineering, University of Chemistry and Technology, Technicka 3, 16628 Prague, Czech Republic; jaroslav.hanus@vscht.cz; 6 Department of Paediatrics and Inherited Metabolic Disorders, First Faculty of Medicine, Charles University and General University Hospital in Prague, Ke Karlovu 2, 12808 Prague, Czech Republic; hana.hansikova@lf1.cuni.cz

**Keywords:** extracellular vesicle, exosome, neurodegenerative disease, Huntington´s disease, huntingtin, fragment, biomarker, pig model, TgHD, KI-HD

## Abstract

(1) Background: Huntington’s disease (HD) is rare incurable hereditary neurodegenerative disorder caused by CAG repeat expansion in the gene coding for the protein huntingtin (HTT). Mutated huntingtin (mHTT) undergoes fragmentation and accumulation, affecting cellular functions and leading to neuronal cell death. Porcine models of HD are used in preclinical testing of currently emerging disease modifying therapies. Such therapies are aimed at reducing mHTT expression, postpone the disease onset, slow down the progression, and point out the need of biomarkers to monitor disease development and therapy efficacy. Recently, extracellular vesicles (EVs), particularly exosomes, gained attention as possible carriers of disease biomarkers. We aimed to characterize HTT and mHTT forms/fragments in blood plasma derived EVs in transgenic (TgHD) and knock-in (KI-HD) porcine models, as well as in HD patients’ plasma. (2) Methods: Small EVs were isolated by ultracentrifugation and HTT forms were visualized by western blotting. (3) Results: The full length 360 kDa HTT co-isolated with EVs from both the pig model and HD patient plasma. In addition, a ~70 kDa mutant HTT fragment was specific for TgHD pigs. Elevated total huntingtin levels in EVs from plasma of HD groups compared to controls were observed in both pig models and HD patients, however only in TgHD were they significant (*p* = 0.02). (4) Conclusions: Our study represents a valuable initial step towards the characterization of EV content in the search for HD biomarkers.

## 1. Introduction

Huntington’s disease (HD) is a rare, progressive, and fatal hereditary neurodegenerative disorder. It is clinically characterized by progressive motor, cognitive, and psychiatric disturbance ending in death within 15–20 years after diagnosis [1,2]. HD is inherited in an autosomal dominant manner and is caused by expanded CAG (Cytosine-Adenine-Guanine) repeats (≥36) in exon I of the huntingtin coding gene (IT15), resulting in expression of the mutant huntingtin protein (mHTT) with an abnormal number of glutamine repeats (polyQ, ≥36) in its N-terminus [3,4]. Huntingtin (HTT) is a large protein (350 kDa) that is ubiquitously expressed in human tissues with highest expression in the central nervous system (CNS) and testes [5]. HTT plays fundamental roles in human development and normal brain function [6], it acts as a scaffolding protein that co-operates with other proteins in various cellular and physiological functions, including vesicular trafficking, transcription, regulation of cell division, metabolism, and apoptosis [5,7]. HD pathogenesis arises from the combination of mHTT acquiring toxic properties, as well as a loss of normal HTT functions [7,8]. The toxic effects of mHTT may be mediated by posttranslational modifications, such as phosphorylation, acetylation, SUMOylation, and ubiquitination, as well as protease cleavage into smaller fragments [9]. The fragments with expanded polyQ repeats are prone to misfold and aggregate into amyloid like structures. The most vulnerable cells to the mHTT toxic effects are specific neurons in the striatum [10]. HD is a multisystem disorder with skeletal muscle atrophy, heart failure, testicular atrophy and immune disturbances being the major symptoms. Disruption of cell metabolism, transcriptional and mitochondrial defects are mostly recognized as contributors to the peripheral tissue pathology [5,11,12,13,14,15,16]. Currently there are no disease modifying treatments for HD available. However, several therapeutic strategies targeting either DNA or RNA coding mHTT are emerging and results from animal models show promise for disease modification in the presymptomatic stage to prevent or delay the onset and slow the disease progression [2,8,17]. However, there is a lack of powerful and noninvasive biomarkers to allow monitoring of the disease particularly in presymptomatic and early symptomatic phases, evaluate the efficacy of the applied treatments, and to design new therapeutic intervention strategies.

Several HD animal models have been generated, including nematode, insect, fish, rodent or large mammals [18,19,20,21,22,23]. The pig models, rather than small mammals, share anatomical, physiological, and pathophysiological similarities with human, and allow the generation of genetically modified models for translational research [22,24,25,26]. Minipigs have adult body weight of 80–110 kg and a long life span (15–20 years), have a gyrencephalic brain with human like neuroanatomy, and blood supply which makes them an ideal representative model of human neurodegenerative disease pathology, progression, and therapeutic interventions [22,27,28]. Various minipig models including HD [25], spinal cord injury [29], and melanoma [30,31] have been generated at our institute. 

Recently, interest has risen in extracellular vesicles (EVs), mainly exosomes, as a probable source for novel non-invasive therapeutic and diagnostic biomarkers for neurodegenerative disorders [32,33,34]. EVs are small bilayer membrane covered vesicles secreted from all cell types into the extracellular environment and body fluids [35,36]. Based on their cellular biogenesis and size, they are classified into three subtypes; exosomes (30–150 nm), microvesicles (100–1000 nm), and apoptotic bodies (50–5000 nm) [37]. Exosomes are the most studied EVs. Exosomes are generated via the inward budding of the early endosomes and the invagination of their membranes to form the intraluminal vesicles (ILVs) within large multivesicular bodies (MVBs). Subsequently, MVBs fuse with the membranes to release exosomes into the surrounding extracellular environment [35,38]. EVs contain a wide range of bioactive molecules including proteins, lipids, messenger RNAs, micro RNAs, and long non-coding RNAs, and metabolites [35,38,39]. They have very important roles in cell–cell communication, homeostasis maintenance, cytokine secretion and transport, which might induce chemotaxis, inflammation or apoptosis, and are engaged in antigen presentation in immune regulation [38,39]. In the CNS, EVs (particularly exosomes) can be secreted from all cell types including neurons, astrocytes, oligodendrocytes, and microglia [40,41,42]. They act as a vehicle for intercellular communication and transport and are involved in synaptic plasticity, glutamate uptake, neuroprotection, and may promote axonal transport and modulate neuroinflammation [34,42,43]. EVs can overcome the blood-brain barrier (BBB) and enter the blood stream, particularly in neurodegenerative diseases and neuroinflammation, accompanied by BBB disruption [32,44,45,46].

In neurodegenerative diseases, neurotoxic misfolded proteins can be selectively integrated into ILVs of MVBs and thence released into the extracellular environment within exosomes [47,48]. Exosomes may spread the misfolded proteins across the CNS [42,49,50,51]. In HD, the spread of mHTT by EVs was reported both in vitro [52] and in vivo [53]. On the other hand, incorporation of misfolded mHTT into EVs may be neuroprotective by reducing intracellular mHTT aggregates [54,55] through their elimination from cells [56]. Due to the ability to cross the BBB, EVs have a great potential in delivery of therapeutics into CNS [57].

EVs and particularly exosomes have a large potential as non-invasive diagnostic biomarker carriers for neurodegenerative diseases and various studies reported them as being a source for biomarkers in Alzheimer’s disease (AD) [58,59,60,61,62], Parkinson’s disease (PD) [63,64,65,66,67], amyotrophic lateral sclerosis (ALS) [50,68,69], and prion disease [48]. The amyloid β (Aβ), tau, α-synuclein, and superoxide dismutase 1 were previously quantified in EVs in AD, PD, and ALS, and in several studies the correlation of their levels with the disease stages were found [51,60,64,70,71,72,73,74]. In HD, information about protein composition of EVs is missing [57] and the presence of HTT protein or its fragments in the EVs isolated from blood plasma have not yet been reported. 

The aim of this study was to map the presence of HTT protein and its mutant and fragmented forms in small EVs (in size corresponding to exosomes) isolated from blood plasma. We compared the HTT forms in plasma-derived EVs of two porcine models of HD, i.e., transgenic HD (TgHD) [25] and knock-in HD (KI-HD) [22] models, as well as in EVs from HD patient plasma. The isolated EVs were characterized using several techniques, including transmission electron microscopy (TEM), nanoparticle tracking analysis (NTA), flow cytometry, and Western blotting. Huntingtin N-terminal as well as polyQ- specific antibodies were used to detect HTT forms and their size on western blot. Finally, we compared HTT forms and their quantities in plasma EVs between HD and control individuals in both pig models and HD patients. 

## 2. Results

### 2.1. Isolation and Characterization of the EVs

**Figure 1:**
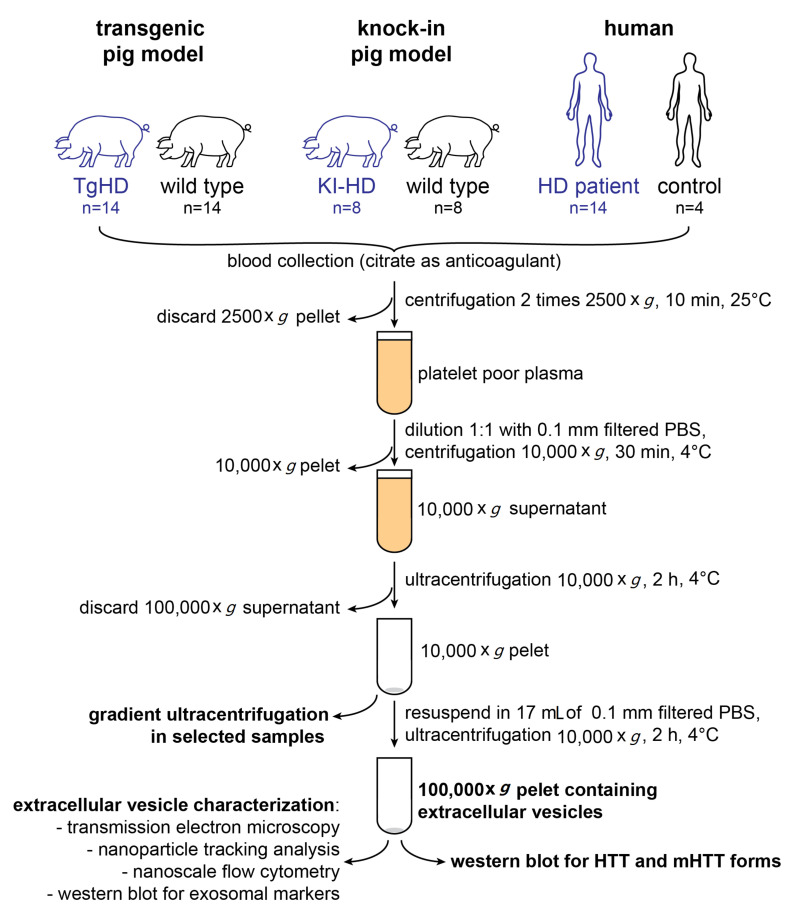
Experimental scheme.

EVs were isolated from blood plasma of TgHD and KI-HD pig models, and HD patients and control plasma using ultracentrifugation (UC) (see Figure 1 for experimental scheme and Section 4.1. for animal model description). Characterization of the small EVs was performed in accordance with MISEV 2018 (minimal information for studies of extracellular vesicles) guidelines [75]. EV size was analyzed in the vast majority of pig samples. However, in case of human samples, the volume of available plasma was not sufficient to obtain EV material for all the methods, including western blots. Thus, the EV size was characterized only in a few human samples with larger plasma volumes available. 

#### 2.1.1. Morphological Characterization of EVs by TEM

**Figure 2:**
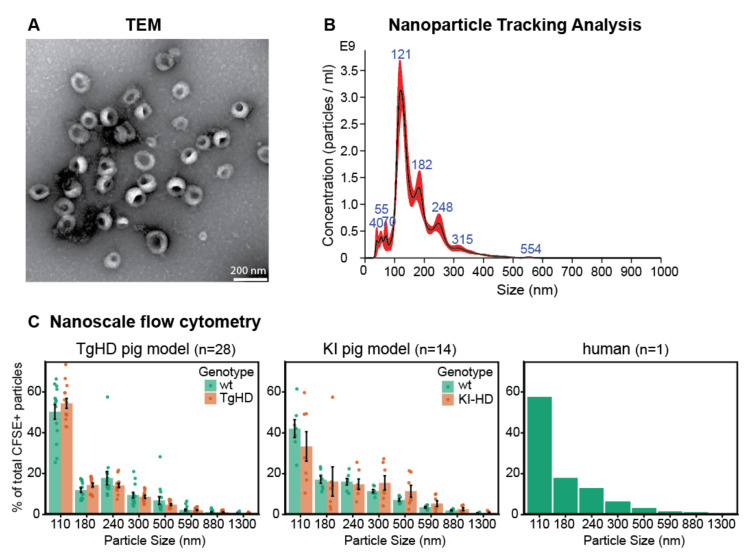
Characterization of the morphology and size of extracellular vesicles. (**A**) TEM confirmed presence of spherical vesicles with diameter smaller than 200 nm in the ultracentrifugation pellets from porcine and human plasma (representative image from 20 samples analyzed by TEM is shown). Magnification 100,000×. (**B**) Characterization of particle size by NTA. Representative result from 46 samples analyzed by NTA is shown. Error bars (red) indicate ±1 standard error of the mean (see Table S1 for detailed data). (**C**) Nanoscale flow cytometry with fluorescent CFSE labelling was used to assess size of particles of biological origin in ultracentrifugation pellets from plasma. The most frequent particle size category was 110 nm. Dots represent individual samples, bars represent mean of % of total CFSE+ particles in individual genotype and size groups, error bars show standard error of the mean (see Table S1 for detailed data).

Size and morphology of EVs isolated from blood plasma were assessed by TEM (Figure 2A). The presence of cup-shaped vesicles with the lipid bilayer membrane and diameter mostly bellow 200 nm was confirmed in 100,000× *g* pellets from both animal model plasma, as well as human blood plasma (Figure S1). In total, 20 samples were analyzed by TEM across both pig models and HD patient samples.

#### 2.1.2. Characterization of Particle Size by NTA

NTA was performed for EVs derived from blood plasma of fourteen TgHD pigs and thirteen of their wild type (WT) controls, seven KI-HD pigs and seven of their WT controls, and one HD patient and four control persons. In a majority of analyzed samples, the most frequent particle size (mode) ranged from 115 nm to 130 nm (Figure 2B and Figure S2, Table S1). There were no significant differences (*p* < 0.05) in the size mode of the nanoparticles among all the analyzed samples (*n* = 46) based on the genotype and age.

#### 2.1.3. Characterization of Particle Size Distribution by Flow Cytometry

Nanoscale flow cytometry was used to assess possible contamination of small EV isolates by larger particles. The particle size analysis was performed in the size range approximately corresponding to 110–1300 nm. Labelling by 5(6)-Carboxyfluorescein diacetate N-succinimidyl ester (CFSE) was used to detect particles of biological origin. The size distribution of the isolated EVs from blood plasma of HD and control individuals from TgHD (*n* = 28) and KI-HD (*n* = 14) models and a human sample are shown in Figure 2C and Table S1. The isolated particles with 110 nm in diameter were dominant in all samples (*n* = 43) from all the experimental animals as well as the human samples. In both TgHD and KI-HD models, there was no statistically significant difference (*p* < 0.05) in the amount of 110 nm particles isolated from plasma of HD and WT pigs.

### 2.2. Detection of HTT, Mutant HTT, and Exosome Markers in UC Pellets

Anti-N-terminal HTT antibody (EPR5526) and a polyQ-specific antibody (MW1) were used on western blots to reveal the presence of the endogenous HTT and mHTT in the plasma-derived EVs. In addition, enrichment of exosomes in the UC pellets from the blood plasma was tested using antibodies against exosome markers Alix, TSG101, and CD9 (in pig samples), or CD9 and CD63 (in human samples). Analyzed exosome proteins were selected based on specific reactivity of antibodies with the given species.

#### 2.2.1. Detection of HTT Forms in UC Pellets from Plasma of the TgHD Model

**Figure 3:**
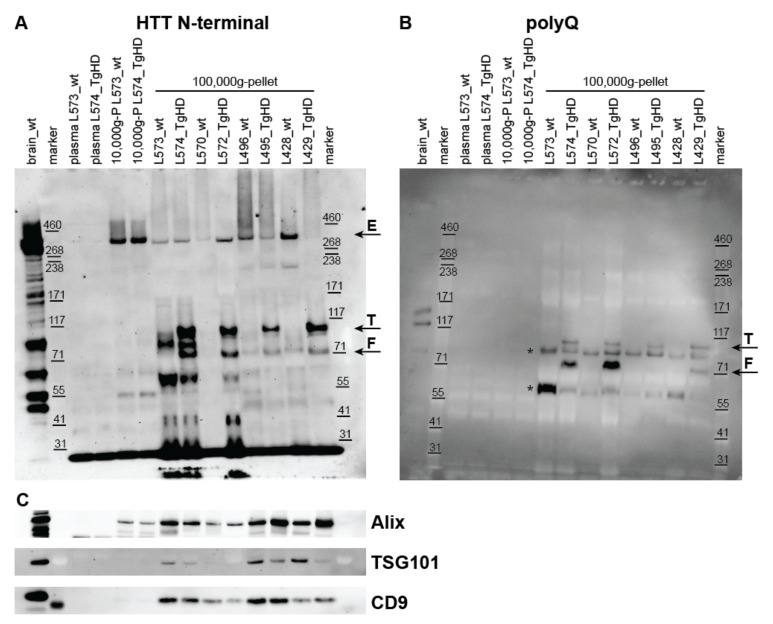
Huntingtin and exosome marker detection in 100,000× *g* centrifugation pellets containing extracellular vesicles, isolated from blood plasma of the transgenic Huntington´s disease pig model. (**A**) anti-N-terminal HTT antibody (EPR5526) was used to detect total HTT levels in plasma, 10,000× *g* pellets and 100,000× *g* pellets. Wild-type pig brain homogenate was used as positive control. Endogenous full length ~360 kDa HTT was detectable in 10,000× *g* and 100,000× *g* pellets from plasma of both wild type (wt) and transgenic (TgHD) pigs (arrow E). In addition, the ~110 kDa human HTT transgene (arrow T) and a ~70 kDa fragment (arrow F) were detectable in TgHD pig 100,000× *g* pellets. (**B**) A polyQ-specific antibody (MW1) provided the same ~110 and ~70 kDa band pattern in TgHD pig 100,000× *g* pellets as EPR5526, thus confirming the presence of mutant HTT (arrows). Additional polyQ-containing protein bands were detected in 100,000× *g* pellets (asterisks). (**C**) Expression of exosome markers Alix, TSG101 and CD9 were abundant in 100,000× *g* pellets containing extracellular vesicles, in contrast to 10,000× *g* pellets and original plasma (sample IDs are identical as in panel (**A**)).

In the TgHD model, HTT N-terminal antibody clearly showed the presence of endogenous full length HTT (~360 kDa) in 10,000× *g* and 100,000× *g* pellets obtained from blood plasma of both TgHD and WT controls (Figure 3A and Figures S3–S5). Moreover, the N-terminal fragment of human mHTT (~110 kDa, corresponding to transgene) and an additional ~70 kDa mHTT fragment were detected in the TgHD minipig samples (Figure 3A) and were absent in WT controls. 

The presence of ~110 and ~70 kDa mHTT bands was confirmed in plasma-derived EVs of TgHD animals and also by a polyQ-specific antibody (Figure 3B and Figures S3–S5). The ~70 kDa band represents mHTT fragment as it is present only in TgHD samples and not in WT controls, and it was detected by both EPR5526 and MW1 antibodies. Additional polyQ protein bands were detected in the 100,000× *g* pellets regardless of the genotype, and as these were not detected using the EPR5526 antibody, they probably represent polyQ containing proteins other than HTT (Figure 3B, asterisk).

#### 2.2.2. Detection of HTT Forms in UC Pellets from Plasma of the KI-HD Model

In the KI-HD model, the endogenous full length HTT (~360 kDa) was detected by the N-terminal HTT antibody in the 10,000× *g* and 100,000× *g* pellets obtained from the blood plasma of both KI-HD minipigs and their WT controls (Figure S6A). Furthermore, an additional band at approximately ~370 kDa was detected in the 100,000× *g* pellets of KI-HD minipig plasma, corresponding to the full length mHTT with expanded polyglutamine repeats (Figure S6A, arrows). Moreover, a very faint band of the full length mHTT (~370 kDa) was detected also in the 10,000× *g* and 100,000× *g* pellets of KI-HD minipig plasma using the anti-polyQ antibody (MW1) (Figure S6B), confirming the presence of mHTT in EVs.

#### 2.2.3. Detection of HTT Forms in UC Pellets from Human Plasma

In EVs isolated from plasma of HD patients and controls, the full length HTT (~360 kDa) showed a similar pattern to that in TgHD and KI-HD minipigs, i.e., the ~360 kDa HTT band present in the 10,000× *g* and 100,000× *g* pellets (Figure S7A). In contrast to the KI-HD model carrying 85 Q, where two forms of HTT (~360 kDa) and mHTT (~370 kDa) could be recognized by the EPR5526 antibody, in humans two such forms could not be distinguished, as the patients carried a lower number of Q repeats in their mHTT (40–47 Q) and both HTT and mHTT showed close to identical electrophoretic mobilities. Similarly, to the KI-HD minipigs, a very faint band of the full length mHTT (~360 kDa) was detected in the 100,000× *g* pellets of HD patients’ plasma using the anti-polyQ antibody (MW1) (Figure S7B, arrows).

#### 2.2.4. Detection of Exosome Markers in UC Pellets

The exosome markers showed abundance in UC pellets containing small EVs in contrast to original platelet-poor plasma (PPP), which confirmed the enrichment of exosomes by UC. In addition, only low amounts of exosome markers were detected in the 10,000× *g* pellets, suggesting that the EVs were not lost during the differential centrifugation step at 10,000× *g* performed to remove possible contaminants such as cell debris and larger vesicles (Figure 3C, Figures S3C–S7C).

### 2.3. Density Gradient UC to Confirm Co-Isolation of HTT and mHTT with Exosomes

The density gradient UC can eliminate non-exosome particles and protein contaminants (e.g., soluble proteins and lipoproteins), maximizing exosome recovery and purity [76]. In our experiment, the OptiPrep density gradient UC was used to confirm and validate the co-isolation of HTT with the small EVs or exosomes, from the blood plasma of TgHD (*n* = 2) and KI-HD (*n* = 2) minipigs, and a human control. The 100,000× *g* pellets from simple UC containing EVs were further separated by the gradient UC in the bottom-up settings in order to allow the vesicles to rise up to the lower OptiPrep densities. A total of seventeen fractions (1 mL each) were collected from top of the tube to the bottom. Equal volumes of all fractions were loaded on gels for western blot to explore the distribution of the endogenous HTT, mHTT, and the exosome markers, among the fractions (OptiPrep densities).

**Figure 4:**
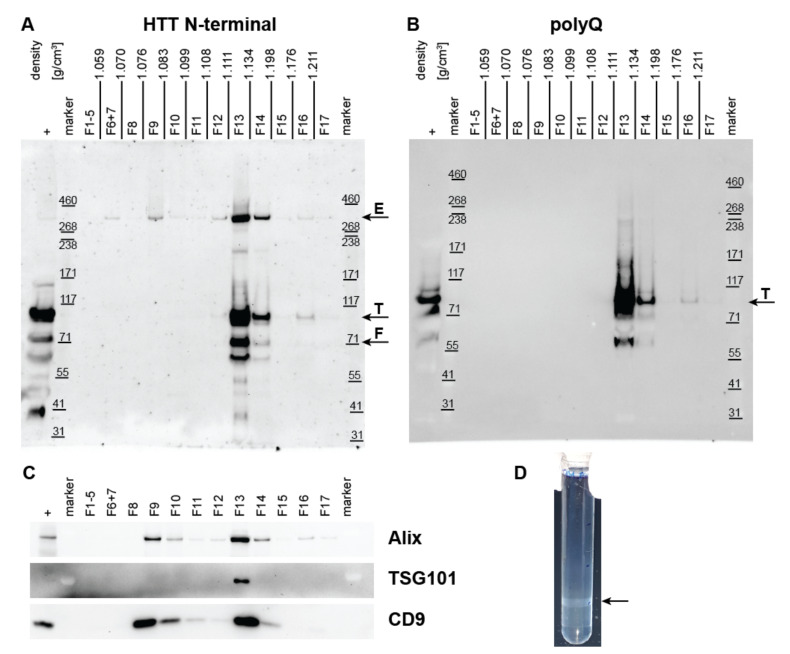
Huntingtin and exosome marker detection in fractions from density gradient ultracentrifugation. The 100,000× *g* pellet from the T131 transgenic pig plasma (sample labeled “+”) was overlaid by 40% to 5% OptiPrep and separated by gradient ultracentrifugation. Seventeen fractions (1 mL each) were collected from top of the tube. The collected fractions were subjected to SDS-PAGE and western blotting to detect total huntingtin (**A**), mutant huntingtin (**B**) and exosome markers Alix, TSG101 and CD9 (**C**). Huntingtin appeared in fraction 13 with density 1.111–1.134 g/cm^3^ corresponding to exosomes. Fraction 13 was also positive to exosome markers. Arrows indicate ~360 kDa endogenous huntingtin (E), ~110 kDa huntingtin transgene (T) and ~70 kDa huntingtin fragment (F). The extracellular vesicles in fraction 13 macroscopically appeared as an opalescent band in the ultracentrifugation tube (arrow in panel (**D**). Representative results from 5 samples fractionated by gradient ultracentrifugation are shown (see Figures S8–S12 for full data).

In TgHD minipigs, all proteins of interest including endogenous HTT (~360 kDa), mHTT (~110 kDa transgene), HTT fragments (~70 and ~60 kDa) and exosome markers (Alix, TSG101, and CD9), appeared in fraction (F) 13, at a density of 1.111–1.136 g/mL (Figure 4, Figures S8 and S9).

In KI-HD minipigs, the full length endogenous HTT (~360 kDa), and the upper band that corresponds to the full length mHTT with expanded polyglutamine repeats (~370 kDa), as well as the exosome markers (Alix, TSG101, and CD9), were detected in F13 and F14, at densities between 1.108 g/mL and 1.122 g/mL (Figures S10 and S11). In both pig models, additional HTT and exosome marker bands occurred in F9-F10 (Figures S8–S11).

In human, due to the limited amount of plasma, the density gradient UC was performed only on one healthy individual sample and was analyzed for the presence of the HTT and exosome markers. Similar to the TgHD and KI-HD pigs, there were exosome markers as well as HTT (~360 kDa), detected in F13 and F14, at a density 1.101–1.169 g/mL, respectively, in the human sample (Figure S12). The gradient UC confirmed the co-isolation of HTT/mHTT with exosomes, as both entities rose up from the tube bottom to fractions 13–14 with exosome-specific density.

### 2.4. Quantification of HTT/mHTT Levels in EVs from TgHD and KI-HD Models and HD Patients

**Figure 5:**
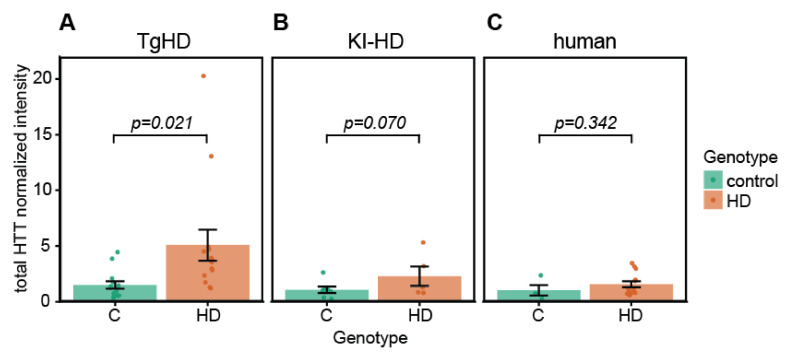
Normalized intensity of total huntingtin in extracellular vesicles isolated from blood plasma of TgHD and KI-HD pig models and human plasma. EVs were isolated by ultracentrifugation and HTT forms were detected by western blot using anti-N-terminal HTT antibody recognizing both normal and mutant HTT. HTT band intensities were quantified by ImageLab software and normalized to CD9 levels to minimize variability in EV loading amount. Significantly higher total HTT content was found in vesicles from TgHD pigs compared to WT control pigs (**A**), while in KI-HD (**B**) and HD patients (**C**) the increase in total HTT in EVs compared to controls was not significant. The dots represent individual samples, bars show mean of the groups, and error bars indicate standard error of the mean. Statistical analysis using three-way ANOVA with genotype and age and sex as factors, followed by the post-hoc Tukey HSD test was performed for pig samples. T-test was used to compare HTT intensity between HD patient and control groups.

The intensities of individual HTT/mHTT bands detected by N-terminal anti-HTT antibody (EPR5526) on western blots as well as CD9 band intensities were quantified (Table S1). To obtain the relative abundance of HTT forms in EVs, the HTT/mHTT band intensities were normalized to the CD9 intensities. An overview of such normalized intensities of individual HTT forms between control and HD groups of TgHD and KI-HD pig models, as well as in human plasma-derived EVs is in Figure S13. Statistical analysis did not reveal any differences in the full length ~360 kDa HTT band intensities in EVs between HD and control groups of TgHD, KI-HD and HD patients. However, the sum of intensities of all HTT/mHTT forms in individual samples showed significantly higher total HTT amounts in EVs from TgHD pigs compared to WT controls (*p* = 0.021) (Figure 5A). Increased total HTT levels in EVs were also detected in the HD group of the KI-HD pig model and HD patient EVs compared to the control group, but the differences were not significant (Figure 5B,C). In both animal models, there were no differences in total HTT levels according to age or sex.

## 3. Discussion

Misfolding, aggregation and accumulation of proteins is a hallmark of neurodegenerative diseases. The protein accumulation and cell damage starts years or even decades before the clinical onset of the disease and clear clinical symptoms are visible only after substantial damage of the brain. The ultimate aim of current therapies is to slow down the disease progression in early symptomatic or possibly pre-symptomatic phases. Thus, improved imaging techniques and sensitive biochemical biomarkers for early and noninvasive diagnosis are urgently needed. The direct detection of misfolded proteins and oligomers in body fluids is challenging as they are present in very low amounts and are highly heterogenous [77]. Nonetheless, several techniques for ultrasensitive HTT detection in body fluids have been introduced, including TR-FRET [78], single molecule counting [79], mesoscale discovery assays [80] and alphaLISA [81], with various sensitivities to normal/mutant HTT and to the oligomer and fragmented HTT forms.

Extracellular vesicles recently captured attention as possible carriers of misfolded proteins in body fluids with diagnostic potential in neurodegenerative disorders. In AD, correlation of phospho-S396-tau, phospho-T181-tau, and Aβ1–42 levels in neural-derived blood exosomes with AD progress was described and, interestingly, in the study of Fiandaca et al., such proteins could predict AD up to 10 years prior to clinical onset [58,60]. Similarly, in PD, substantially higher levels of α-synuclein in neural-derived blood exosomes were identified in a large cohort of PD patients compared to healthy controls [63]. In HD, the presence of HTT in body fluid derived EVs has not yet been reported. Thus, we aimed to detect HTT, mHTT and their fragments in blood plasma EVs. We compared the presence and quantity of HTT/mHTT forms in plasma EVs originating from two pig models of HD and human patients. The pig models were involved in the study as these are unique large animal models used for development and testing of HD modifying therapies [17,82] and enable blood collection in higher amounts to obtain sufficient vesicles for method optimization and detection antibody testing.

Plasma was chosen as it is easily collected and contains circulating EVs released from various cell types throughout the body, including the brain derived EVs that are supposed to cross the BBB. Despite this, we expect that brain-derived EVs represent only a tiny minority of plasma vesicles. However, we did not use enrichment of neural-derived EVs by the L1CAM antibodies as done in other studies in AD and PD [60,63], to keep the isolation protocol simple and reproducible, particularly when two different species were included in our study. In addition, unlike Tau protein and α-synuclein, which have prevalent expression in brain, the HTT is expressed throughout the body and HD affects peripheral organs also [83,84]. Thus, not only the central nervous system, but also other organs can be sources of EVs of interest.

The major population of EVs in blood are platelet-derived EVs and these are present in lower amounts in plasma compared to serum [85]. In our experiment, we minimized the platelet activation and EV release using several measures. The blood was collected by 20 G needle directly to citrate-containing tubes, blood was immediately centrifuged at room temperature to prevent temperature shocks and platelet activation and in order to quickly remove blood cells. A total of two prolonged centrifugation steps were used to obtain platelet poor plasma. Interestingly, despite the platelet-derived EVs forming a majority of blood EVs and despite the platelets contain the highest amounts of mHTT among all blood cells [86], the mHTT was undetectable in platelet-derived EVs [87]. 

Several methods are used to isolate EVs from different biological fluids and tissues such as UC, density gradient UC, size exclusion chromatography, antibody capturing and polymer based precipitation [88]. UC is the current gold standard in the isolation of exosomes in sufficient amount and purity, and despite its low throughput, high time demands and high tube costs, it is the most widely used technique [89,90,91]. In our experiment, we used differential centrifugation followed by UC, washing and additional UC to increase the yield and the purity of small EVs [92]. Our results indicate the enrichment of exosome markers (Alix, TSG101, and CD9) in the UC pellet obtained from HD and healthy controls and across the experimental groups (TgHD, KI-HD, and HD patients), compared to the original PPP and 10,000× *g* pellet. At the same time, larger contaminating vesicles (corresponding in size to apoptotic bodies and larger microvesicles) were mostly removed, as confirmed by TEM, NTA analysis and nano-flow cytometry. This is in agreement with our previous study, where the protocols for small EV isolation by UC from pig blood plasma, seminal plasma and cerebrospinal fluid were described [93]. 

Western blotting was chosen for the detection of HTT/mHTT forms as it can clearly distinguish full length protein and its fragments and provides valuable information about fragment size (molecular weight). The control samples were loaded on the gel according to the total protein amount (5 µg), while the UC pellet containing EV samples were loaded by volume (10 µL of the lysate corresponding to 1 mL of the original plasma, with roughly 2–15 µg of total proteins). We decided to load the EV lysates by volume rather than by protein amount, as the total protein values determined by BCA assay differed largely between individual samples, probably due to presence of the residual soluble plasma proteins (e.g., albumin). Loading by volume ensured less variability in EV loaded amount among samples. Additional washes of UC pellets in PBS might remove more soluble plasma proteins, however, we preferred only one wash to prevent extensive loss of EVs.

Antibodies for total (EPR5526) as well as mutated (MW1) HTT were used to confirm mHTT presence in western blot bands. The MW1 antibody provided clearly visible mHTT bands in the TgHD model, but very faint detection in KI-HD and human samples (at 370–360 kDa), despite optimization of conditions. This is probably caused by the different polyQ lengths in individual HD groups (145 Q in TgHD, 85 Q in KI-HD and 40–47 Q in HD patients) leading to a higher number of antibody molecules attached to polyQ in TgHD samples giving a higher signal. Specifically, in TgHD pig samples, we could clearly identify a ~70 kDa N-terminal fragment of mHTT, in parallel, with both antibodies. Such TgHD pigs carry the human mHTT transgene (expressing ~110 kDa mHTT N-terminal fragment) in addition to two normal pig HTT alleles. The ~70 kDa fragment is a product of fragmentation of the ~110 kDa human mHTT N-terminal fragment, as it is present only in EVs from TgHD pigs and not from their WT siblings. Interestingly, in a study on HeLa cells, the 70 kDa N-terminal HTT was produced from full length HTT after DNA damage and caspase-3 activation and was relocated to nuclei [94]. In several KI-HD animals, a band specific for the mHTT (~370 kDa; 85 Q) was detectable in addition to the wt HTT (~360 kDa; ~18 Q). Additional anti-HTT antibodies have been tested (N-terminal MAB5490 and MAB2166; polyQ-specific 1C2; data not shown) for HTT detection in EVs, but the EPR5526 and MW1 gave the best reactivity and comparable results in both species. A total of three exosome markers were analyzed in all EV samples by western blot in pig samples and two markers in human samples to confirm enrichment of small EVs in UC pellets. 

Importantly, the density gradient UC was used to further separate particles and molecules present in the UC pellet and to increase the purity of EVs [91,95]. The co-isolation of HTT/mHTT with EVs was clearly visible from the western blots of individual gradient fractions, where HTT/mHTT and exosome markers appeared within the same fractions. The density of such fractions was in the range 1.101–1.206 g/mL in five replicates of the gradient UC, which is consistent with the EV densities from 1.094 to 1.287 g/mL previously reported for cell culture supernatant [90,91,96,97], rat plasma [98] or human plasma/serum [88,95] derived EVs. On the other hand, proteins, including possible free HTT aggregates (not associated with EVs) are supposed to stay in the bottom of the tube (F17). In F17, HTT/mHTT was practically undetectable, suggesting that HTT is associated with EVs rather than present as free aggregates in plasma. 

Finally, we attempted to quantify the amounts of individual HTT and mHTT band intensities in EVs between HD and control groups. There were no statistical differences in the full length ~360 kDa HTT band intensities between HD and control groups in any of the analyzed pig models and HD patients. However, if we count the sum of the intensities of all HTT forms in EVs, significantly higher amounts of total HTT were identified in TgHD compared to WT control samples (*p* = 0.021). Higher HTT amounts can be influenced by different HTT coding gene dose between TgHD and WT control pigs. A non-significant increase in total HTT was also found in plasma-derived EVs from KI-HD pigs and HD patients compared to appropriate controls. 

We proved that HTT co-isolates with EVs from blood plasma, identified ~70 kDa mHTT fragment in EVs of TgHD pigs and found significantly higher total HTT levels in TgHD pig plasma derived EVs compared to WT controls. Our study represents a valuable initial step for further characterization of molecular composition of EVs in HD in the search for biomarkers for monitoring disease development and therapy efficacy, or mHTT spread. Importantly, our results open a new way for subsequent studies that might, for example, focus specifically on tissue (e.g., neural) specific EVs in body fluids and/or apply ultrasensitive methods to quantify HTT, other proteins, RNAs or other molecules in EVs in the search for HD biomarkers.

## 4. Materials and Methods

### 4.1. Experimental Animals

All animal experiments were performed in agreement with the Animal Care and Use Committee of the Institute of Animal Physiology, under the Czech regulations and guidelines for the care and use of experimental animals (Approval for use of experimental animals, No. 71922/2016-MZE-17214, issued by Ministry of Agriculture of the Czech Republic) and approved by the Inter-resort committee of the Czech Academy of Sciences (Projects of Experiments 75/2017 and 65/2018).

There were two porcine models included in this study. The first was the TgHD Libechov minipig model that was generated by microinjection of HIV1-HD-548aaHTT-145 Q lentiviral vector into porcine embryos [25] in the PIGMOD research center and Institute of Animal Physiology and Genetics, Czech Academy of Sciences. The TgHD pigs express the N-terminal part of human mHTT (N-terminal 548 amino acids with 124 Q) under the control of the human HTT promoter, corresponding to approx. 110 kDa HTT protein N-terminal fragment [25,99]. In this model, the expression of mHTT was detected at both the mRNA and protein level in all tissues with the highest levels in the brain and testes as well as in blood and cerebrospinal fluid (CSF). The TgHD minipigs carry both endogenous porcine alleles coding the wild type HTT in addition to the human mHTT fragment and such a gene dose lengthens the presymptomatic stage of the disease [5,100]. The motor, cognitive and behavioral decline accompanied by signs of neurodegeneration on histological and biochemical levels were documented in TgHD pigs at the age of 5–8 years of age [28,101]. Currently, the PIGMOD center breeds TgHD and their WT siblings with identical genetic backgrounds which are subjected to phenotypic evaluation as well as sponsored preclinical studies of HTT lowering therapies [22]. TgHD animals involved in the study belonged to two age groups, i.e., 2-year old (*n* = 8) and 7-year old (*n* = 20).

The second model are the KI-HD minipigs. This model was established by Cure Huntington’s Disease Initiative foundation (CHDI) at Exemplar Genetics and is currently bred at the PIGMOD center. The CAG tract was expanded by adeno associated virus mediated homologous recombination in minipig fibroblasts followed by somatic cell nuclear transfer. The KI-HD heterozygotes carrying one wild type HTT and one mHTT with elongated polyQ (85 Q) were used in our study. In this model, the full length mHTT is expressed in all tissues as well as in blood and CSF [22]. Animals involved in the study were 6 months old (*n* = 2), 12 months old (*n* = 8) and 18 months old (*n* = 6). As the KI-HD model has been established recently, older animals were not available at the time of the study. Details about the animals of both models used in the study are in Table S1.

### 4.2. HD Patients

The HD patients and healthy controls were enrolled to the study at the Department of Neurology and Center of Clinical Neuroscience of the 1st Faculty of Medicine, Charles University, and the General University Hospital in Prague. The study was approved by the Ethical committee of the General University Hospital (protocol No. 6/18). The selection of patients was random, regardless of gender, at different stages of the disease and with different numbers of CAG triplets. The patient data are summarized in Table S1. 

### 4.3. Blood Sample Collection and PPP Preparation

Venous blood samples (30 mL) were collected from fasting TgHD minipigs (*n* = 14) and their WT controls (*n* = 14) and from KI-HD minipigs (*n* = 7) and their WT controls (*n* = 7) by venipuncture of the vena cava cranialis using 50 mL syringes (20 G needle) pre-filled with isotonic sodium citrate buffer, pH 7.4 (final citrate concentration 0.5%) as an anticoagulant. Blood samples were immediately centrifuged at 2500× *g* for 15 min at room temperature (RT) to remove cellular components, and to prevent platelet activation and release of platelet derived extracellular vesicles. Supernatant was transferred into a clean tube and centrifuged again at 2500× *g* for 15 min at RT. Supernatant (PPP) was cooled to 4 °C prior to being used for UC. Aliquots of 0.2 mL PPP were stored at −80 °C and used as controls for western blots.

In the HD patients, the blood (15 mL) was collected from the cubital vein by a 20 G needle into citrate vacuum tubes. The patients´ samples were processed in the same way as the pig samples, only after the second 2500× *g* centrifugation the PPP was frozen at −80 °C.

### 4.4. Isolation of Extracellular Vesicles

#### 4.4.1. UC

Extracellular vesicles were isolated from blood plasma as previously described by Théry et al. [102], with modifications from recent protocols [92,93]. Briefly, PPP (17 mL in pigs, 8 mL in human samples) was diluted 1:1 by 0.1 µm filtered ice-cold phosphate buffer saline (PBS) to decrease sample viscosity, transferred to 17 mL polypropylene UltraClear ultracentrifuge tubes (Beckman Coulter, Brea, CA, USA) and centrifuged at 10,000× *g* (8800 rpm) for 30 min at 4 °C using a SW32.1 rotor (k-factor 229) in an Optima L-90K ultracentrifuge (Beckman Coulter). The resultant pellets were resuspended in 100 µL of filtered ice-cold PBS and stored at −80 °C for further analyses. The resultant supernatant was transferred to a new 17 mL polypropylene UltraClear ultracentrifuge tube and centrifuged at 100,000× *g* (28,000 rpm) for 130 min at 4 °C using a SW32.1 rotor. The supernatant was then discarded, and the pellets were resuspended in 17 mL of filtered ice-cold PBS and centrifuged again at 100,000× *g* for 70 min at 4 °C using the same tubes and rotor. The supernatant was discarded, and the EV pellets were resuspended in 150 µL PBS and stored at −80 °C for further analysis. Aliquots of the EV suspension were directly used for NTA (5 µL), flow cytometry (5 µL) and TEM (2 µL). As smaller amount of blood was collected in patients and to minimize sample loss, the 100,000× *g* pellets were directly lysed in ice-cold 1× concentrated radioimmunoprecipitation assay buffer (RIPA) (final concentration in lysate 150 mM NaCl, 5 mM EDTA, 50 mM Tris HCl, 0.5% NP-40, 1% sodium deoxycholate, 1% Triton X-100, 0.1% SDS at pH 7.4) with 1× concentrated HALT™ protease and phospahatase inhibitor cocktail (ThermoFisher Scientific, Waltham, MA, USA) for western blot in the human samples. In several human samples, the 100,000× *g* pellets were resuspended in PBS and used for TEM, NTA and flow cytometry to characterize the isolated EVs. 

#### 4.4.2. OptiPrep™ (Iodixanol) Density Gradient UC

A discontinuous iodixanol gradient was constructed according to Tauro et al. [96], as modified by Van Deun et al. [90]. Briefly, a stock solution of OptiPrepTM (60% (*w*/*v*) aqueous iodixanol from Axis-Shield) was diluted with working buffer (0.25 M sucrose, 6 mM EDTA (pH 8.0), 60 mM Tris HCl (pH 7.4)) to obtain 50% (*w*/*v*) iodixanol working solution. Solutions of 40% (*w*/*v*), 20% (*w*/*v*), 10% (*w*/*v*) and 5% (*w*/*v*) iodixanol were made by mixing proper volumes of the 50% iodixanol working solution and homogenization media (0.25 M sucrose, 1 mM EDTA (pH 8.0), 10 mM Tris HCl (pH 7.4)). Prior to the gradient preparation, the pellet from the 100,000× *g* UC was resuspended in 1 mL of 40% (*w*/*v*) iodixanol. The gradients were formed bottom-up by carefully overlaying 1 mL of the sample resuspended in 40% (*w*/*v*) by 3 mL of 40% iodixanol, followed by 4 mL of 20%, 4 mL of 10 %, and finally 4.8 mL of 5% iodixanol in a 17 mL polypropylene UltraClear ultracentrifuge tube (Beckman Coulter). The gradient was then centrifuged at 100,000× *g* (28,000 rpm) overnight at 4 °C using the SW32.1 rotor. Gradient fractions, 1 mL each, were collected from the top of the gradient. To remove iodixanol, the 1 mL factions were diluted with 16 mL filtered ice-cold PBS and centrifuged at 100,000× *g* for 70 min at 4 °C (SW32.1 rotor). The resultant pellets were resuspended in 100 µL ice-cold 1× concentrated RIPA buffer with HALT inhibitor cocktail and stored at −80 °C for further analysis. The density of each fraction was estimated by using an analytical balance scale to determine the weight of each 1 mL fraction. 

### 4.5. Extracellular Vesicle Lysate Preparation

Porcine extracellular vesicle (EV) pellets from 100,000× *g* UC resuspended in PBS were mixed with an equal volume of 2× concentrated RIPA lysis buffer with HALT inhibitor cocktail and incubated for 10 min on ice with occasional vortexing. The lysates were sonicated for 1 min at 4 °C and centrifuged at 16,000× *g* for 10 min at 4 °C to remove insoluble debris. The resulting supernatant was then transferred into a new Eppendorf tube and stored at −80 °C for further analysis. The 10,000× *g* pellet aliquots were lysed and processed in the same way. The PPP aliquots were diluted (50 times) in a volume of 1× RIPA with HALT inhibitor lysis buffer to obtain 0.5–3 mg/mL protein concentration in lysates and were then processed as EV lysates. Protein concentration in the lysates was determined by the BCA protein assay (ThermoFisher Scientific) according to manufacturer’s protocol using bovine serum albumin standards. Lysates of human neural stem cells and porcine tissues were used as controls for western blotting and were prepared as described previously [93,103].

### 4.6. Western Blot Analysis

A total of five micrograms of total proteins in control sample lysates (human neural stem cells, porcine whole tissues, PPP, and 10,000× *g* pellets) and 10 µL of EV lysates (corresponding to EVs obtained from 1 mL of original PPP) were mixed with lithium dodecyl sulfate sample buffer and 50 mM dithiothreitol (DTT) (Invitrogen), heated for 5 min at 70 °C and loaded onto precast 3–8% Tris-acetate or 4–12% bis-tris NuPAGE™ polyacrylamide minigels (Invitrogen), and separated at 150 V for 90 min in Tris-acetate and MES running buffers, respectively. After electrophoresis, proteins were electrotransferred onto nitrocellulose membranes using the semidry Trans-Blot Turbo Transfer System (Bio-Rad, Hercules, CA, USA). Depending on the primary antibody used, membranes were blocked with 5% (*w*/*v*) skimmed milk or 5% (*w*/*v*) bovine serum albumin (BSA) in Tris-buffered saline with 0.05% Tween 20 (TTBS-T) for 1 h at RT. Membranes were incubated with primary antibodies (see details below) overnight at 4 °C, washed 3 times in TTBS-T for 10 min and then incubated with the corresponding Horseradish Peroxidase-conjugated secondary antibodies (see details below) for 1 h at RT. Membranes were then washed in TTBS-T (3 times, 10 min), incubated with enhanced chemiluminescence reagent (ECL Prime Western Blotting Detection Reagent, GE Healthcare, Chicago, IL, USA) and visualized using the ChemiDocTM XRS+ imaging system (Bio-Rad). 

For HTT detection, anti-HTT N-terminal antibody (EPR5526, Abcam, Cambridge, UK, ab109115, 1:2000 in 5% milk) and anti-PolyQ (MW1, Sigma Aldrich, St. Louis, MO, USA, MABN242, 1:500 in 5% milk) were used. For exosome marker detection, anti-Alix antibody (Abcam, ab88388, 1:1500 in 5% milk), anti-TSG 101 antibody (Santa Cruz Biotechnology, Dallas, TX, USA, sc-7964, 1:200 in 5% milk), anti-CD63 antibody (Santa Cruz Biotechnology, sc-5275, 1:1000 in 5% milk), and anti-CD9 antibody (Abcam, ab92726, 1:500 in 5%BSA) were used. HRP conjugated secondary antibodies (anti-rabbit or anti-mouse IgG, Jackson ImmunoResearch, West Grove, PA, USA, 711-035-152 and 715-035-151, respectively) were diluted 1:10,000 in 5% milk.

### 4.7. TEM

Two microliters of EV UC pellets resuspended in PBS were applied on formvar-carbon coated 400 mesh copper TEM grids, and allowed to adsorb for 30 min. The grids were then incubated in of 2% paraformaldehyde in PBS for 20 min, followed by 6 washes in ultrapure water (2 min each). To contrast the samples, 15 µL of 2% uranylacetate were applied on the grid and incubated for 12 min on ice in the dark, briefly washed in ultrapure water and allowed to dry. Images were acquired on a TEM JEOL 1011 (Tokyo, Japan) equipped with a Veleta CCD camera and Olympus Soft Imaging Solution acquisition software 4.5. 

### 4.8. NTA

The size and concentration of EVs were estimated by NTA using the NanoSight NS300 analyzer (Malvern Instruments, Malvern, UK), equipped with a green laser (<60 mW at 532 nm), sCMOS camera, NTA 3.4 Build 3.4.003 software, and automated syringe pump system. Prior to the NTA measurement, an aliquot (5 µL) of the isolated EVs were diluted in cold 0.1 µm filtered PBS to achieve a measured particle concentration of 25–30 particles per frame. For each sample, five 60-s videos were recorded with camera level and detection threshold set to 14. For analysis, the following settings were selected: detected threshold 5, automatic blur size, automatic maximum track length, and automatic maximum expected particle size.

### 4.9. Nanoscale Flow Cytometry

The flow cytometry was used as described previously [93,104]. The EVs diluted by 0.1 µm filtered PBS were labelled with CFSE (eBioscience, San Diego, CA, USA) according to the manufacturer’s protocol. The Apogee A50-Micro flow cytometer (Apogee Flow Systems, London, UK) was calibrated using a mixture of 180, 240, 300, 590, 880 and 1300 nm silica beads, and 110 and 500 nm green, fluorescent polystyrene beads (Apogee Flow Systems). The number of CFSE-positive particles were calculated in individual size clusters, after subtraction of background (CFSE labelled dilution buffer) as described in [93].

### 4.10. Data Analysis and Statistical Analysis

The ~360 kDa, ~370 kDa, ~110 kDa, ~70 kDa and ~60 kDa HTT/mHTT band intensities in UC pellets of appropriate models detected by the EPR5526 antibody on western blots, as well as the CD9 band intensities, were quantified using ImageLab software (Bio-Rad) on blot exposure images with unsaturated pixel intensities. The band intensities were determined after subtraction of background by the lane and bands analysis tool in ImageLab. The relative intensity of HTT bands was then normalized to the CD9 band intensity in individual samples to minimize any variations in UC pellet resuspension efficacy or unequal sample loading on gels. The molecular weight analysis tool in ImageLab was used to determine the molecular weight of individual bands.

The statistical analysis was performed in the R statistical environment [105] using the tidyverse package [106]. A two-way ANOVA followed by Tukey’s HSD test were used to compare NTA particle size mode in TgHD and KI-HD models, with genotype and age as factors, without interaction. The same statistics were used to compare the amount of 110 nm-sized vesicles analyzed by flow cytometry between TgHD and KI-HD pigs and appropriate WT controls. The particle size in human samples was not statistically evaluated due to a limited number of analyzed samples.

Similarly, three-way ANOVA, with genotype and age and sex as factors, was used to evaluate differences in total HTT (EPR5526 360 kDa + 110 kDa + 70 kDa + 60 kDa bands) normalized intensity between TgHD pigs and their WT controls, as well as in total HTT (EPR5526 360 kDa + 370 kDa bands) normalized intensity between KI-HD pigs and their WT controls. Total HTT (EPR5526 ~360 kDa band only) intensity was compared between HD patients and control human samples using an unpaired *t*-test.

## 5. Conclusions

We reported co-isolation of full length endogenous HTT as well as its mutant forms with EVs from blood plasma. To increase the impact of the study, the HTT forms were compared in two large animal models of HD used in preclinical studies of HTT-lowering therapies as well as in HD patients´ plasma. Significant differences in HTT amounts in plasma derived EVs were found between TgHD pigs and WT controls. Our study represents a first step in the characterization of molecular composition of EVs in HD in the search for valuable biomarkers. However, involvement of larger patient cohorts is required to obtain more reliable results. Moreover, additional studies are needed to further characterize molecular composition, such as protein/RNA/miRNA/lipid content, of either EVs in general or of tissue specific (e.g., neural-derived) EVs. Such studies have potential to identify new HD biomarkers allowing monitoring of the disease development and efficacy of currently emerging therapies, particularly in presymptomatic or early symptomatic phase, with the ultimate aim to slow down the progression of this devastating disease.

## Data Availability

Not applicable.

## Supplementary Materials

The following supporting information can be downloaded at: https://www.mdpi.com/article/10.3390/ijms23105598/s1.
