# Supplementary material for: Huntingtin Co-Isolates with Small Extracellular Vesicles from Blood Plasma of TgHD and KI-HD Pig Models of Huntington’s Disease and Human Blood Plasma
Source: Int J Mol Sci. 2022 May 17;23(10):5598. doi: 10.3390/ijms23105598 (PMC9147436; doi:10.3390/ijms23105598)
Supplement: Supplementary file 1 [file ijms-23-05598-s001.zip › SupplementaryFigures_R1.pdf]

Supplementary Figures

List of Supplementary Figures:

Figure S1
Transmission electron microscopy of extracellular vesicles isolated from plasma of TgHD and KI-HD models and human plasma.

Figure S2
Nanoparticle tracking analysis of extracellular vesicles isolated from plasma of TgHD and KI-HD models and human plasma.

Figure S3
Huntingtin and exosome markers in EVs from plasma of 2-year-old TgHD pigs and their wild type siblings.

Figure S4 (displayed in the main text as Figure 3)
Huntingtin and exosome markers in EVs from plasma of 7-year-old TgHD pigs and their wild type siblings.

Figure S5
Huntingtin and exosome markers in EVs from plasma of 7-year-old TgHD boars and wild type controls.

Figure S6
Huntingtin and exosome markers in EVs from plasma of 6 to 18 month-old KI-HD pigs and their wild type siblings.

Figure S7
Huntingtin and exosome markers in EVs from plasma of Huntington´s disease patients and control persons.

Figure S8 (displayed in the main text as Figure 4)
Huntingtin and exosome markers in EVs separated by density gradient ultracentrifugation in 2-year old TgHD pig.

Figure S9
Huntingtin and exosome markers in EVs separated by density gradient ultracentrifugation in 7-year old TgHD pig.

Figure S10
Huntingtin and exosome markers in EVs separated by density gradient ultracentrifugation in 12-months old KI-HD pig.

Figure S11
Huntingtin and exosome markers in EVs separated by density gradient ultracentrifugation in 18-months-old KI-HD pig.

Figure S12
Huntingtin and exosome markers in EVs separated by density gradient ultracentrifugation of control human plasma.

Figure S13
Normalized intensities of individual huntingtin bands in EVs from plasma of TgHD and KI-HD models and human plasma.

Figure S14
Overview of main findings of the study.

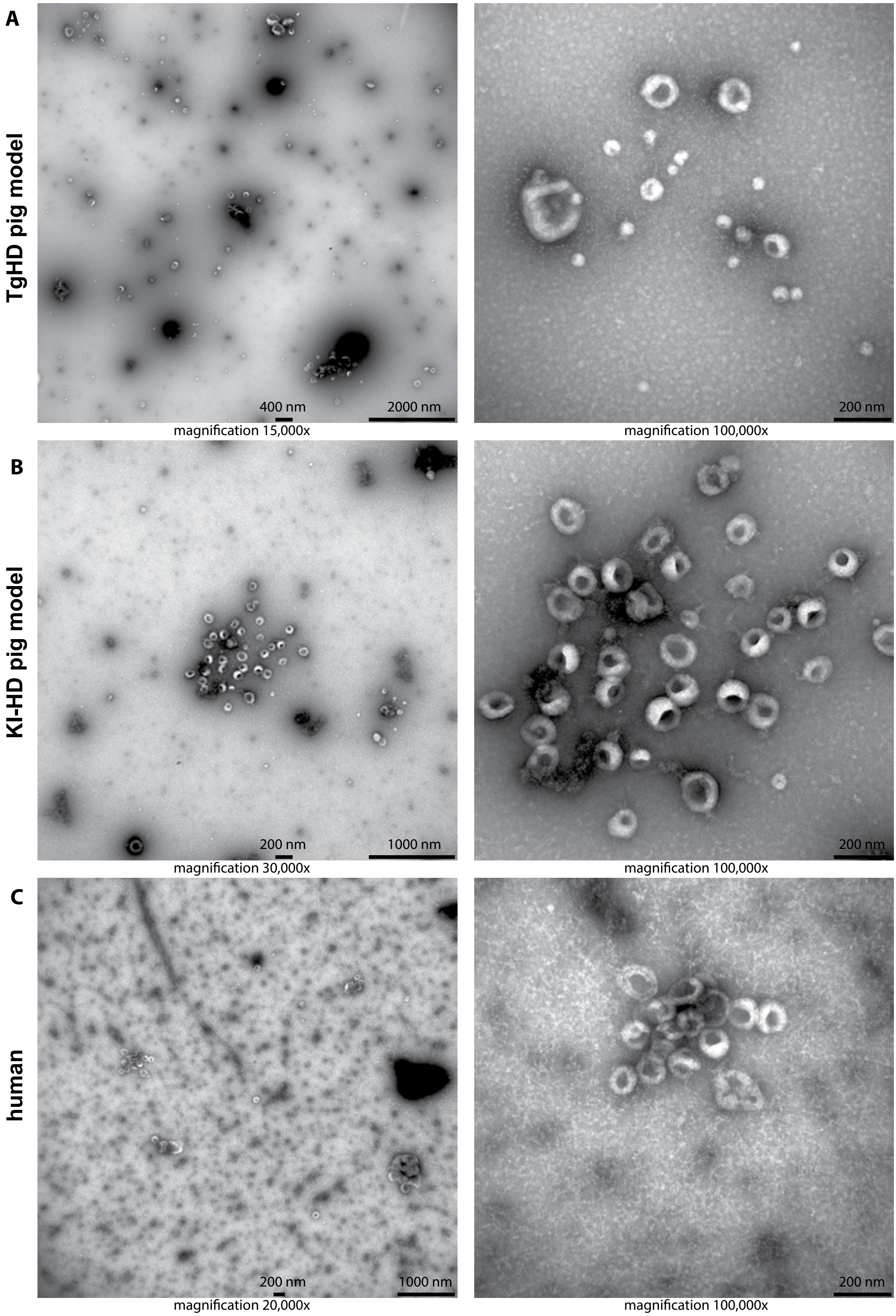

Figure S1

Transmission electron microscopy of extracellular vesicles isolated from plasma of TgHD and KI-HD models and human plasma. Pellet from 100,000 g ultracentrifugation was resuspended in PBS, fixed, contrasted by uranylacetate and visualized by TEM. Vesicles isolated from TgHD pig plasma **(A)**, KI-HD pig plasma **(B)** and human plasma **(C)** had diameter mostly bellow 200 nm. Wide-field images are shown on the left, while zoom-in images are on the right.

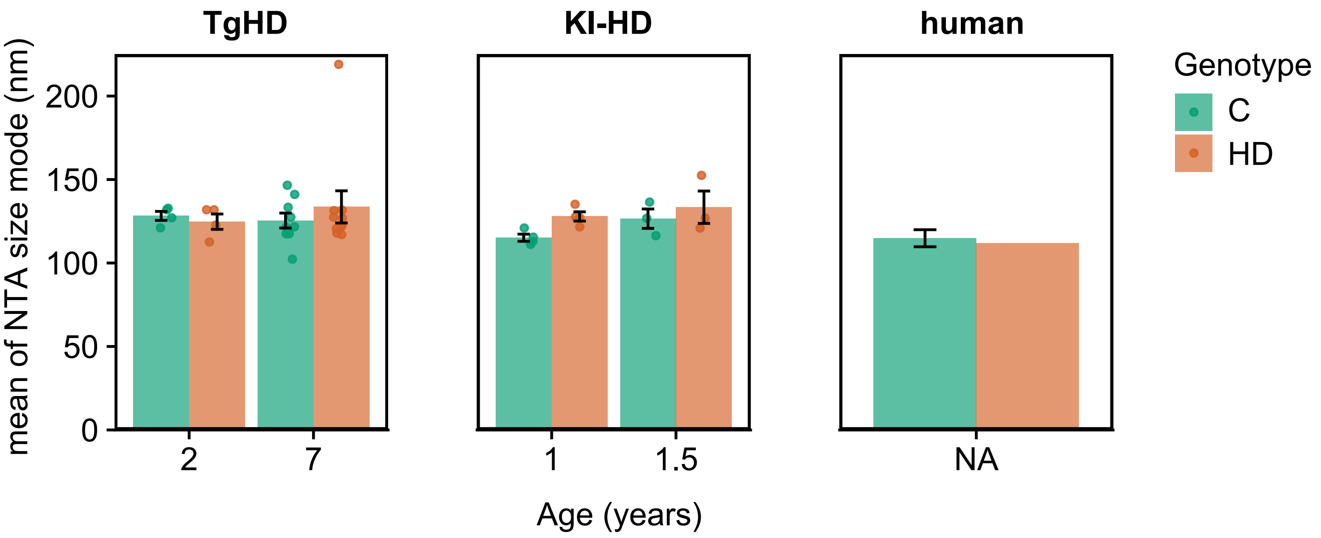

Figure S2

Nanoparticle tracking analysis of extracellular vesicles isolated from plasma of TgHD and KI-HD models and human plasma. The plots show the nanoparticle size mode, i.e. the size that occurs most frequently, of the particles (extracellular vesicles) isolated from plasma of transgenic (n=27) and knock-in (n=14) pig models and human (n=5). The bars indicate mean of the group, the dots denote individual samples. There was not statisticaly significant difference ($p<0.05$) in size mode of the nanoparticles between Huntington´s disease and control samples as well as between animal age groups (2-way ANOVA, with Genotype and Age as factors, without interaction, followed by TukeyHSD test).

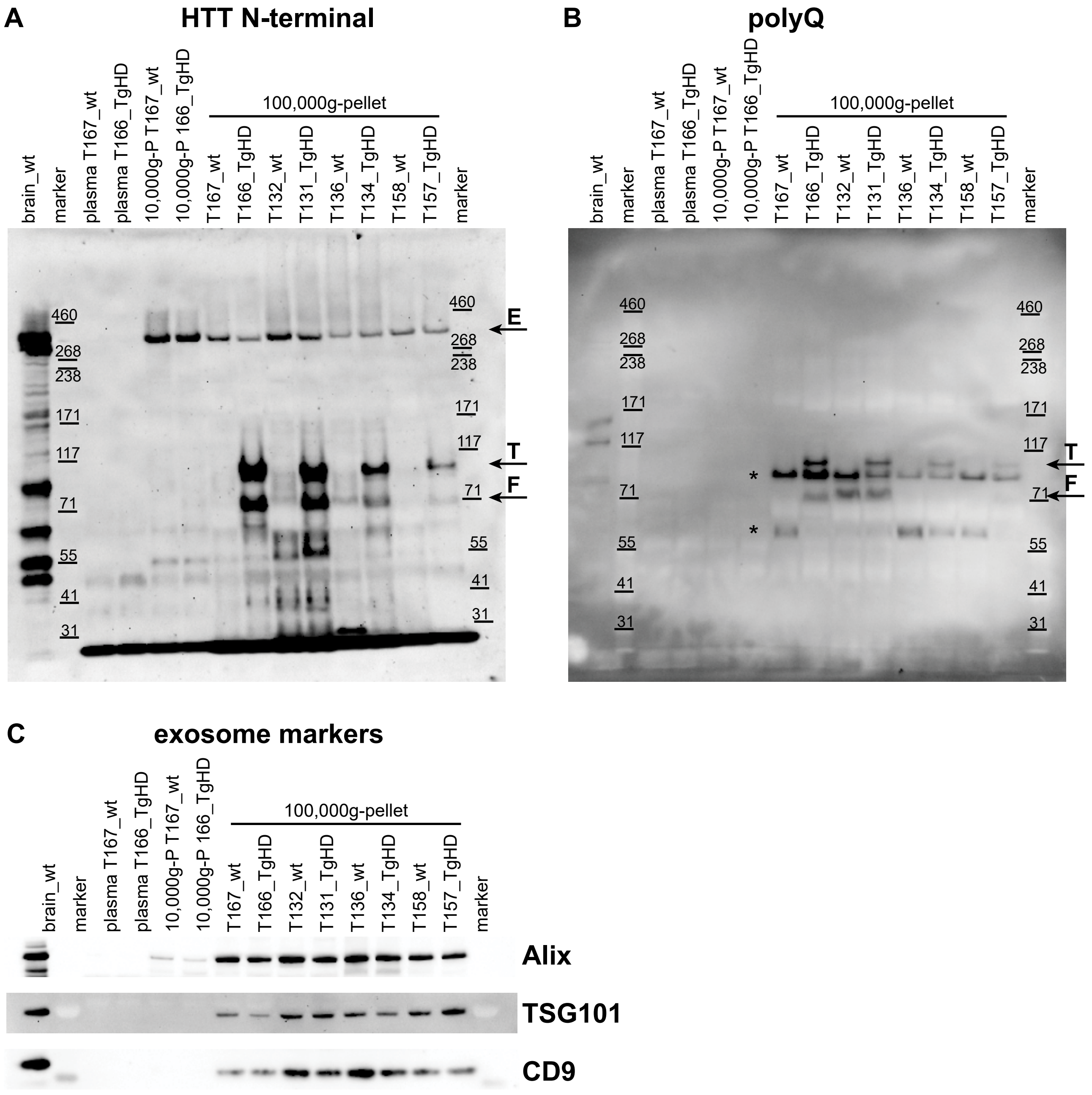

Figure S3

Huntingtin and exosome markers in EVs from plasma of 2-year-old TgHD pigs and their wild type siblings.

(A) Anti-N-terminal HTT antibody (EPR5526) was used to detect total HTT levels in plasma, 10,000g pellets and 100,000g pellets. Endogenous full length ~360 kDa HTT was detectable in 10,000 and 100,000 g pellets from plasma of both wild type (wt) and transgenic (TgHD) pigs (arrow *E*). In addition, the ~110 kDa human HTT transgene (arrow *T*) and a ~70 kDa fragment (arrow *F*) were detectable in TgHD pig 100,000g pellets. **(B)** A polyQ-specific antibody (MW1) provided the same ~110 and ~70 kDa band pattern in TgHD pig 100,000 g pellets as EPR5526, thus confirming the presence of mutant HTT (arrows). Additional polyQ-containing protein bands were detected in 100,000g pellets (asterisks). **(C)** Expression of exosome markers Alix, TSG101 and CD9 was abundant in 100,000g pellets containing extracellular vesicles, in contrast to 10,000 g pellets and original plasma, confirming enrichment of EVs by ultracentrifugation.

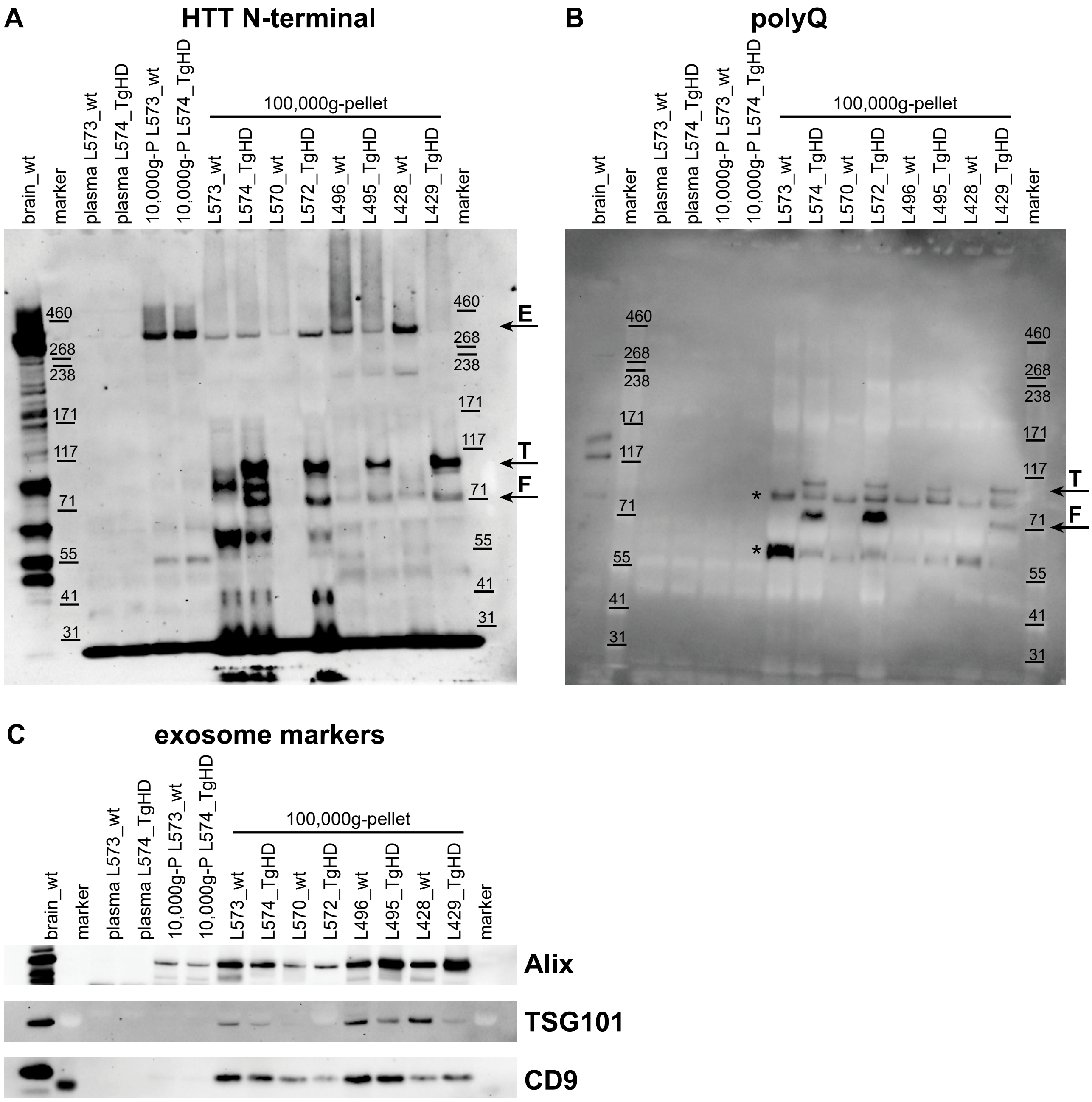

Figure S4 (displayed in the main text as Figure 3)

Huntingtin and exosome markers in EVs from plasma of 7-year-old TgHD pigs and their wild type siblings.

(A) Anti-N-terminal HTT antibody (EPR5526) was used to detect total HTT levels in plasma, 10,000g pellets and 100,000g pellets. Endogenous full length ~360 kDa HTT was detectable in 10,000 and 100,000 g pellets from plasma of both wild type (wt) and transgenic (TgHD) pigs (arrow *E*). In addition, the ~110 kDa human HTT transgene (arrow *T*) and a ~70 kDa fragment (arrow *F*) were detectable in TgHD pig 100,000g pellets. **(B)** A polyQ-specific antibody (MW1) provided the same ~110 and ~70 kDa band pattern in TgHD pig 100,000 g pellets as EPR5526, thus confirming the presence of mutant HTT (arrows). Additional polyQ-containing protein bands were detected in 100,000g pellets (asterisks). **(C)** Expression of exosome markers Alix, TSG101 and CD9 was abundant in 100,000g pellets containing extracellular vesicles, in contrast to 10,000 g pellets and original plasma, confirming enrichment of EVs by ultracentrifugation.

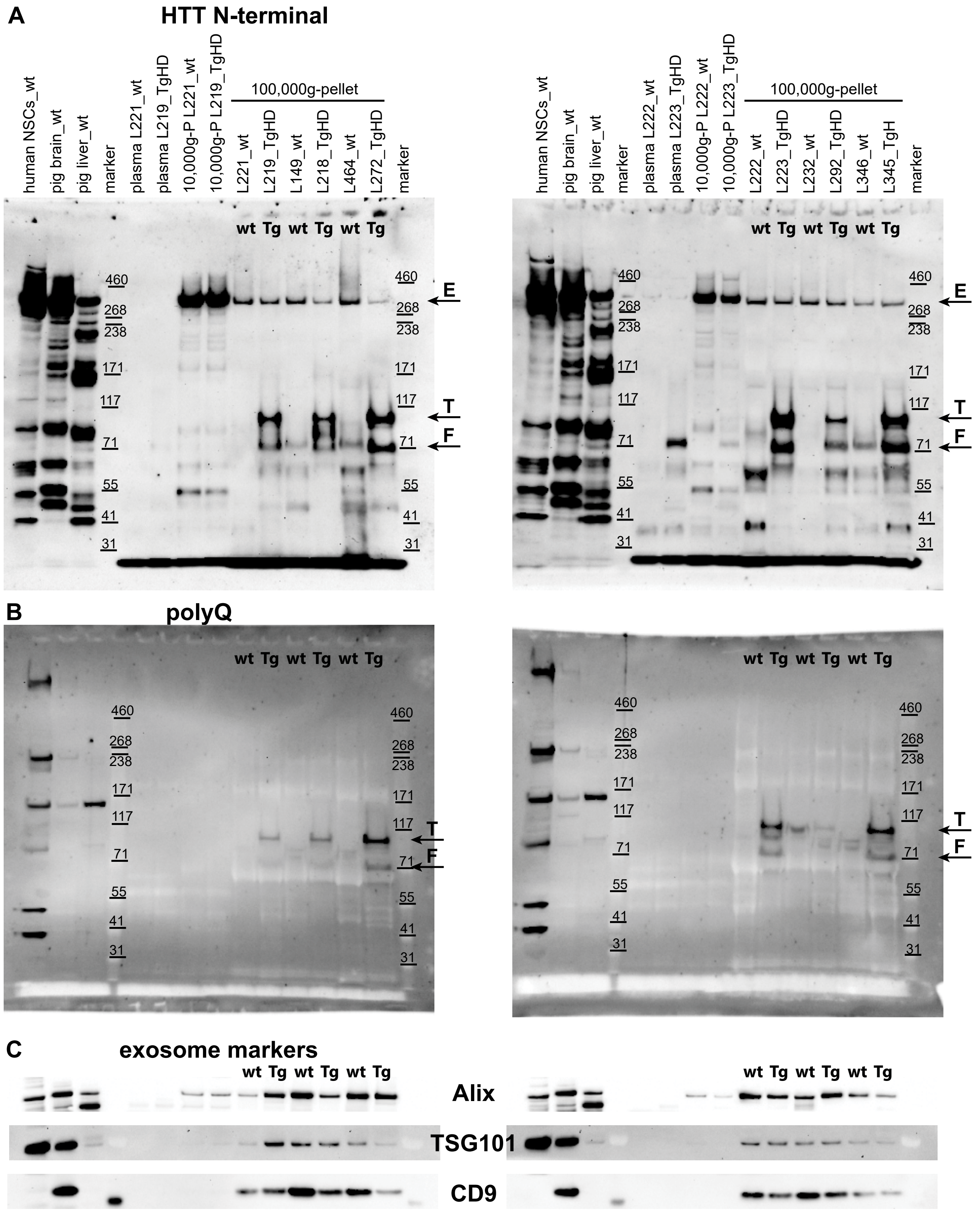

Figure S5

Huntingtin and exosome markers in EVs from plasma of 7-year-old TgHD boars and wild type controls.

(A) Anti-N-terminal HTT antibody (EPR5526) was used to detect total HTT levels in plasma, 10,000g pellets and 100,000g pellets. Endogenous full length ~360 kDa HTT was detectable in 10,000 and 100,000 g pellets from plasma of both wild type (wt) and transgenic (TgHD) pigs (arrow *E*). In addition, the ~110 kDa human HTT transgene (arrow *T*) and a ~70 kDa fragment (arrow *F*) were detectable in TgHD pig 100,000g pellets. **(B)** A polyQ-specific antibody (MW1) provided the same ~110 and ~70 kDa band pattern in TgHD pig 100,000 g pellets as EPR5526, thus confirming the presence of mutant HTT (arrows). **(C)** Expression of exosome markers Alix, TSG101 and CD9 was abundant in 100,000g pellets containing extracellular vesicles, in contrast to 10,000 g pellets and original plasma, confirming enrichment of EVs by ultracentrifugation. In panels B and C, samples were loaded in the identical order as in panel A.

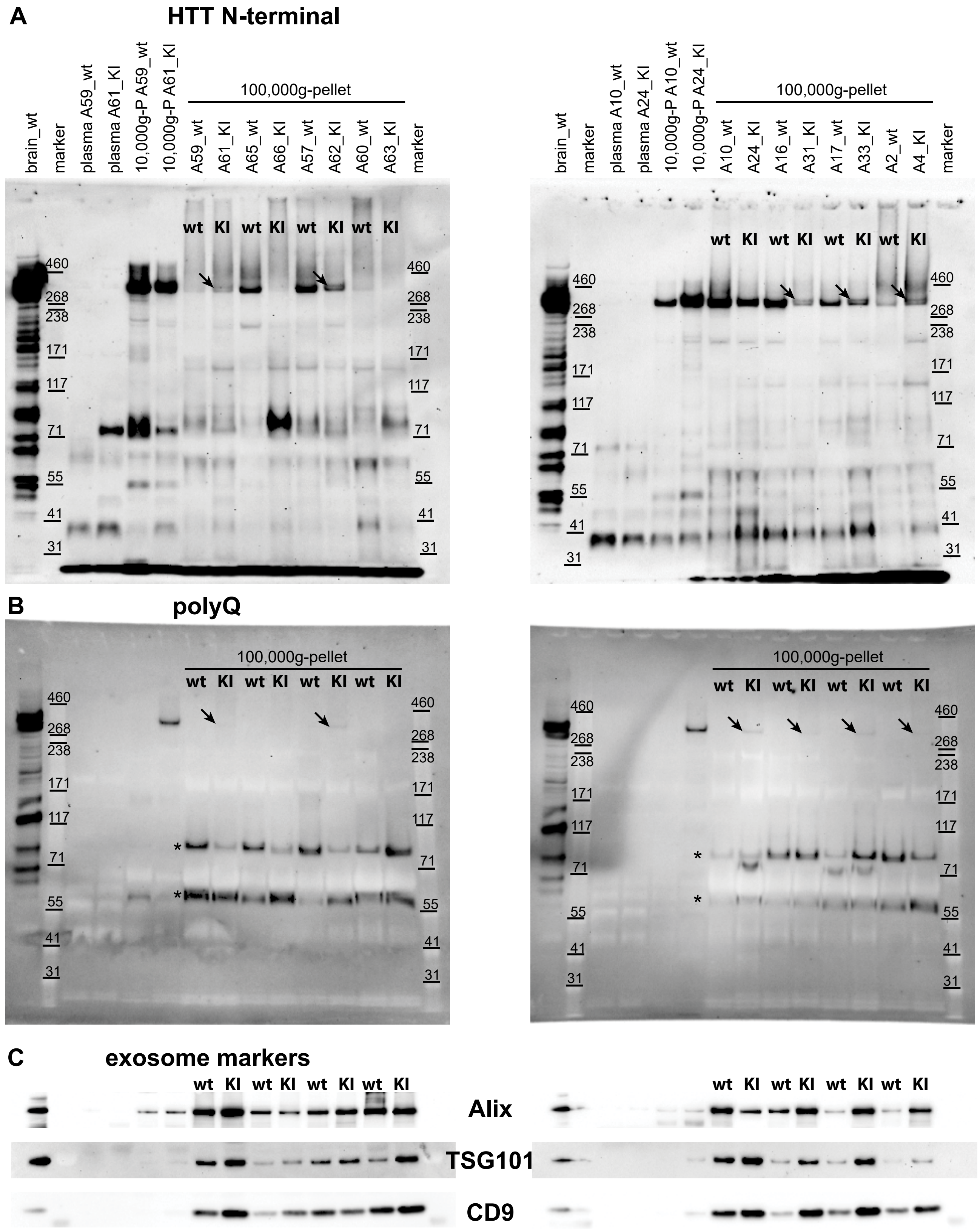

Figure S6

Huntingtin and exosome markers in EVs from plasma of 6 to 18 month-old KI-HD pigs and their wild type siblings.

(A) Anti-N-terminal HTT antibody (EPR5526) was used to detect total HTT levels in plasma, 10,000g pellets and 100,000g pellets. Wild type pig brain homogenate was used as positive control. Endogenous full length ~360 kDa HTT was detectable in 10,000 and 100,000 g pellets from plasma of both wild type (wt) and knock-in (KI) pigs. An additional band corresponding to full length mutant HTT (~370 kDa) was detected in KI-HD samples (arrows). **(B)** Using anti-polyQ-specific antibody (MW1), a very faint band of the full length ~370 kDa mHTT was detected (arrows) in the 100,000 g pellets from plasma of KI-HD animals. Additional polyQ-containing protein bands were detected in 100,000g pellets (asterisks). **(C)** Expression of exosome markers Alix, TSG101 and CD9 was abundant in 100,000g pellets containing extracellular vesicles, in contrast to 10,000 g pellets and original plasma, confirming enrichment of EVs by ultracentrifugation. In panels B and C, samples were loaded in the identical order as in panel A.

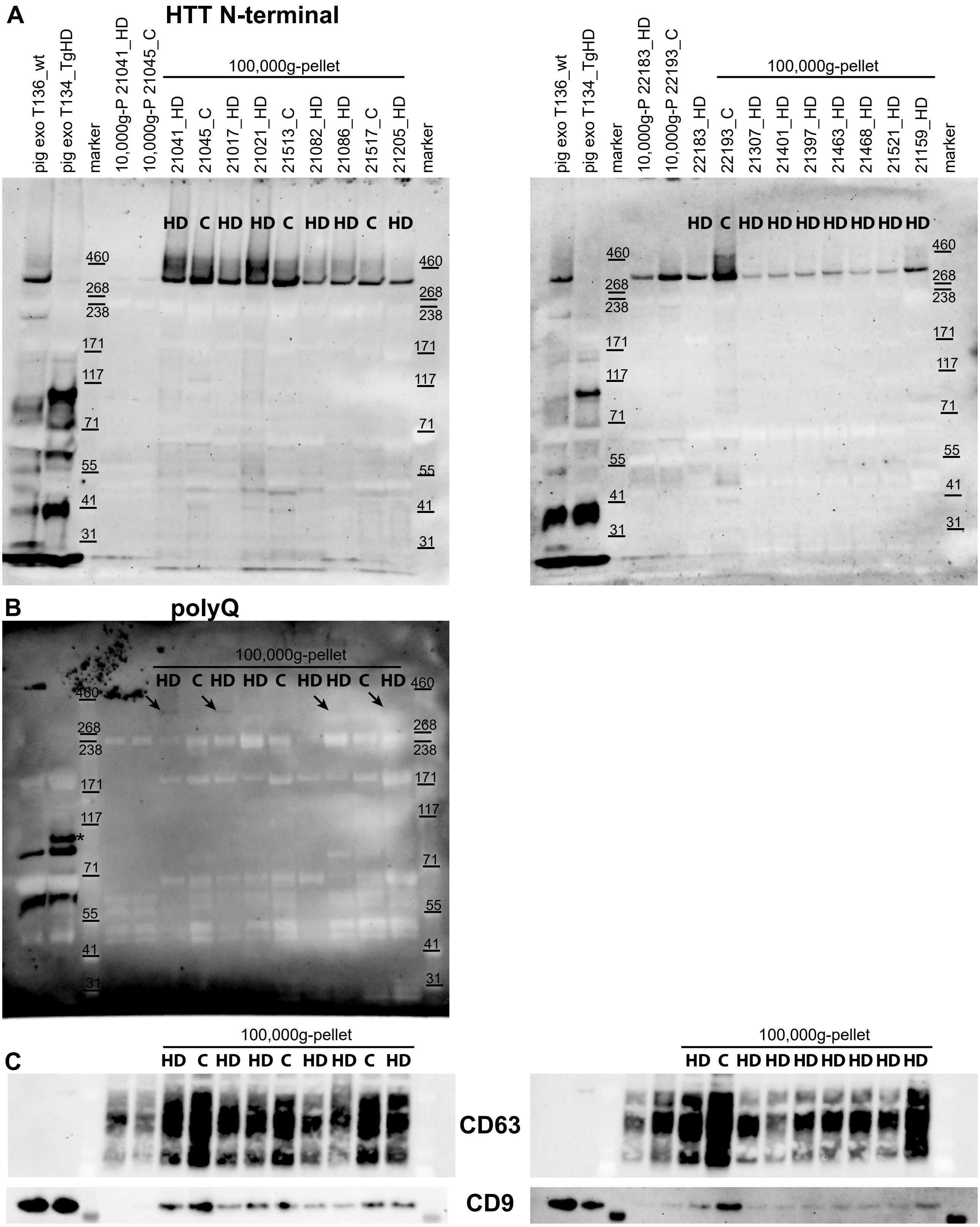

Figure S7

Huntingtin and exosome markers in EVs from plasma of Huntington´s disease patients and control persons.

(A) Anti-N-terminal HTT antibody (EPR5526) was used to detect total HTT levels in 10,000 g and 100,000g pellets. Lysates of 100,000 g pellet of the TgHD pig model plasma were used as a control. Endogenous full length (~360 kDa) HTT was detectable in 10,000 and 100,000 g pellets from plasma of both Huntington´s disease patients and heatlhy controls. **(B)** Using anti-polyQ-specific antibody (MW1), a very faint band of the full length (~360 kDa) mutant HTT was detected (arrows) in the 100,000 g pellet from plasma of HD patients. The asterisk denotes position of the ~110 transgene in TgHD EV sample. **(C)** Expression of exosome markers CD63 and CD9 was abundant in 100,000 pellets containing extracellular vesicles, in contrast to 10,000 g pellets, confirming enrichment of EVs by ultracentrifugation. The CD63 antibody does not recognize porcine CD63.

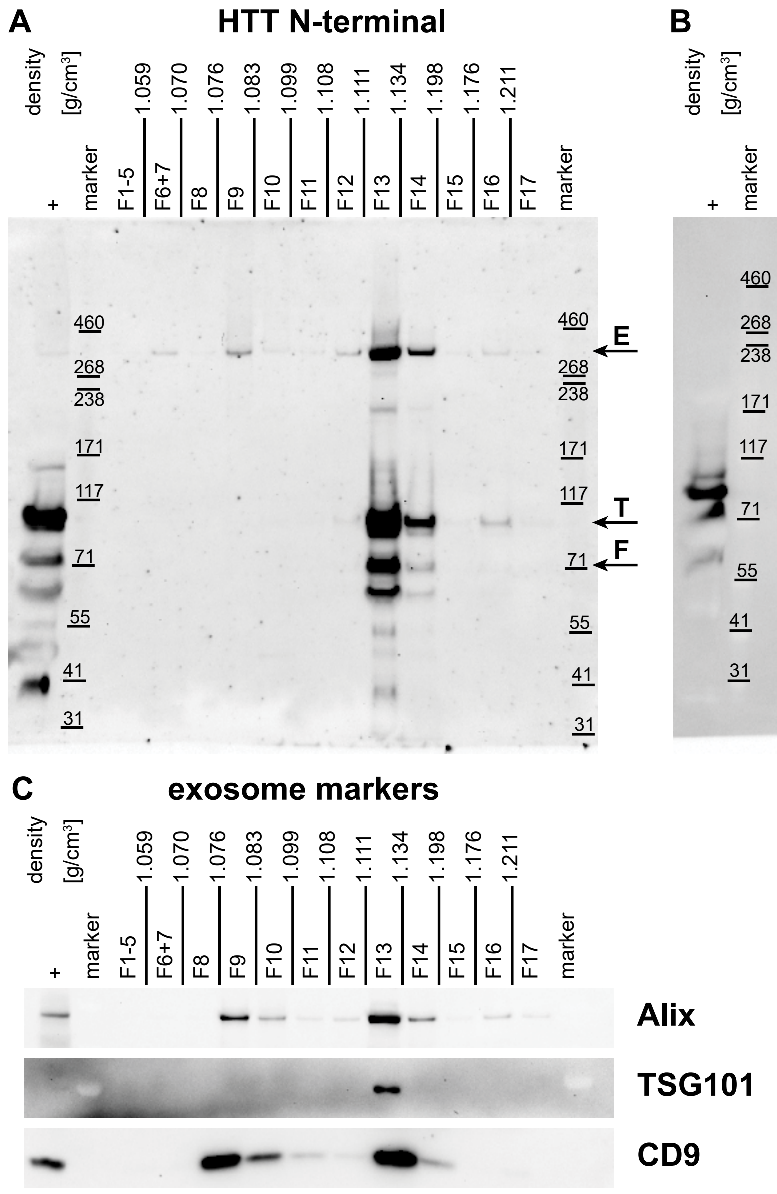

Figure S8 (displayed in the main text as Figure 4)

Huntingtin and exosome markers in EVs separated by density gradient ultracentrifugation in 2-year old TgHD pig.

The 100,000 g pellet from the T131 transgenic pig plasma (sample labeled +) was overlaid by 40% to 5% Optiprep and separated by gradient ultracentrifugation. Seventeen fractions (1 ml each) were collected from top of the tube. The collected fractions were subjected to SDS-PAGE and western blotting to detect total huntingtin **(A)**, mutant huntingtin **(B)** and exosome markers Alix, TSG101 and CD9 **(C)**. Huntingtin appeared in fraction 13, which has the density 1.111-1.134 corresponding to exosomes and was also positive to exosome markers. Arrows indicate ~360 kDa endogenous huntingtin (*E*), ~110 kDa transgenic huntingtin (*T*) and ~70 kDa huntingtin fragment (*F*).

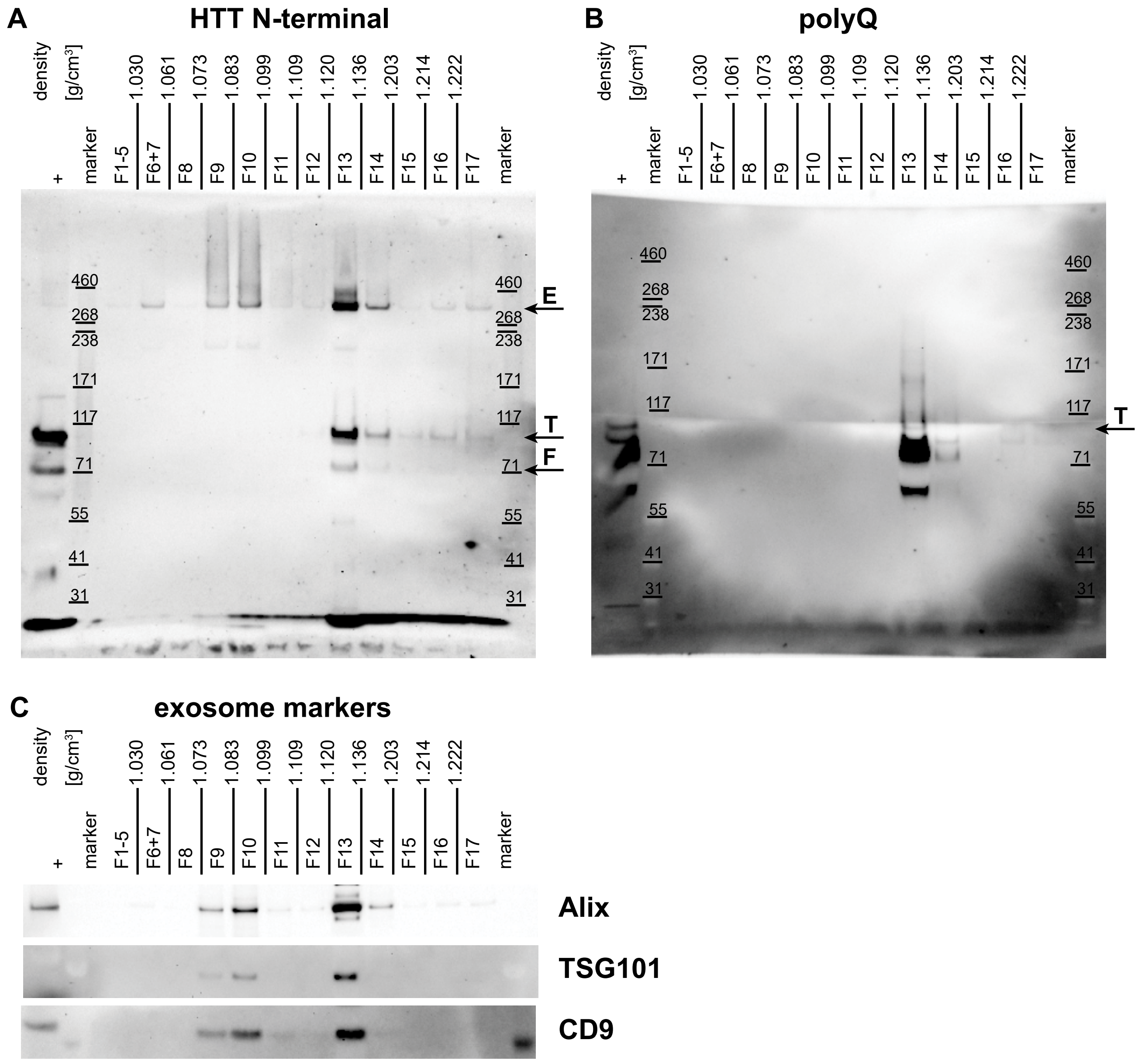

Figure S9

Huntingtin and exosome markers in EVs separated by density gradient ultracentrifugation in 7-year old TgHD pig.

The 100,000 g pellet from the L574 transgenic pig plasma (sample labeled +) was overlaid by 40% to 5% Optiprep and separated by gradient ultracentrifugation. Seventeen fractions (1 ml each) were collected from top of the tube. The collected fractions were subjected to SDS-PAGE and western blotting to detect total huntingtin **(A)**, mutant huntingtin **(B)** and exosome markers Alix, TSG101 and CD9 **(C)**. Huntingtin appeared in fraction 13, which has the density 1.120-1.136 correspoding to exosomes and was also positive to exosome markers. Arrows indicate ~360 kDa endogenous huntingtin (*E*), ~110 kDa transgenic huntingtin (*T*) and ~70 kDa huntingtin fragment (*F*).

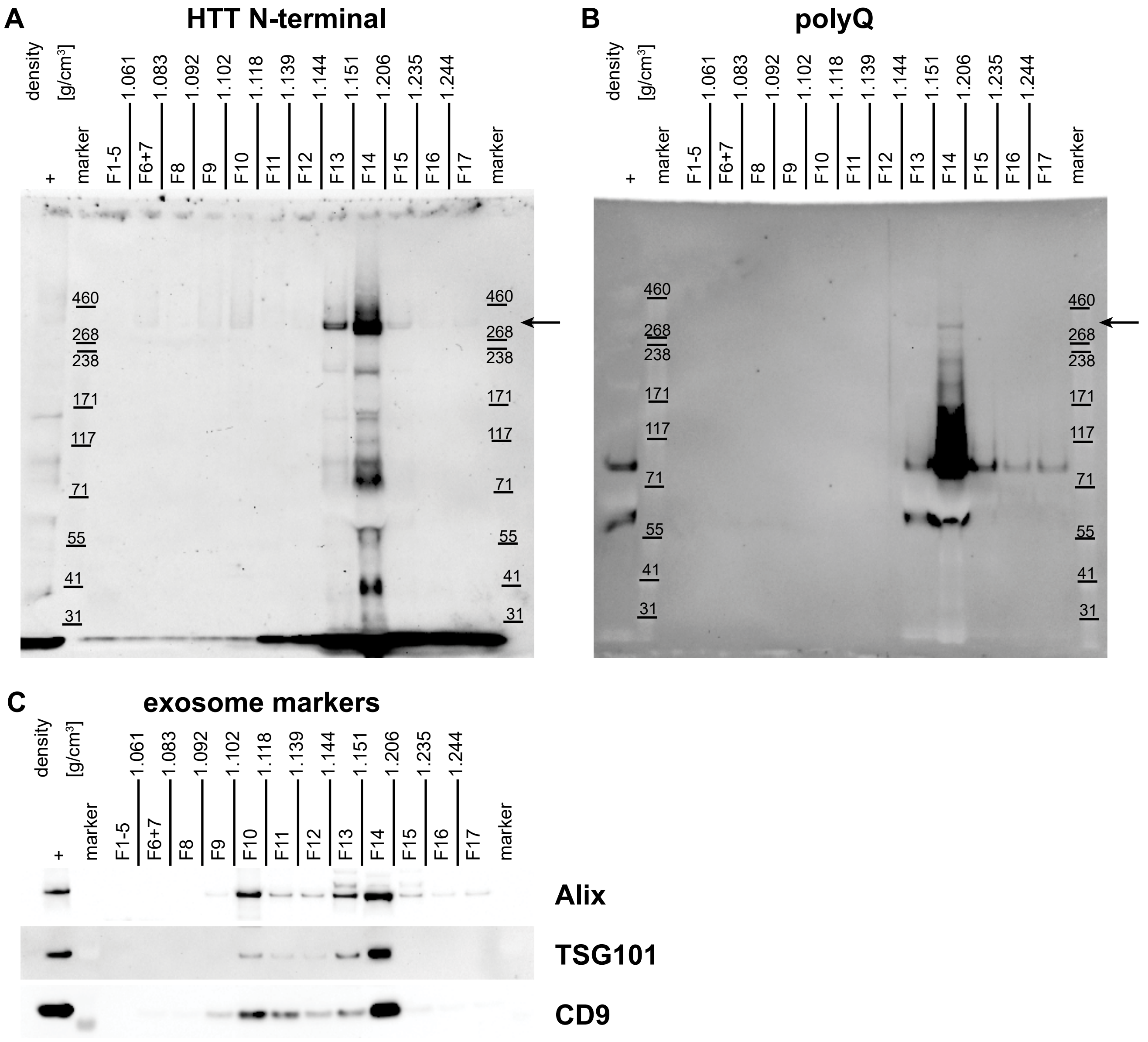

Figure S10

Huntingtin and exosome markers in EVs separated by density gradient ultracentrifugation in 12-months old KI-HD pig. The 100,000 g pellet from the A63 knock-in pig plasma (sample labeled +) was overlaid by 40% to 5% Optiprep and separated by gradient ultracentrifugation. Seventeen fractions (1 ml each) were collected from top of the tube. The collected fractions were subjected to SDS-PAGE and western blotting to detect total huntingtin **(A)**, mutant huntingtin **(B)** and exosome markers Alix, TSG101 and CD9 **(C)**. Huntingtin appeared in fraction 14, which has the density 1.151-1.206 correspoding to exosomes and was also positive to exosome markers. Arrow indicates ~360 kDa full length huntingtin.

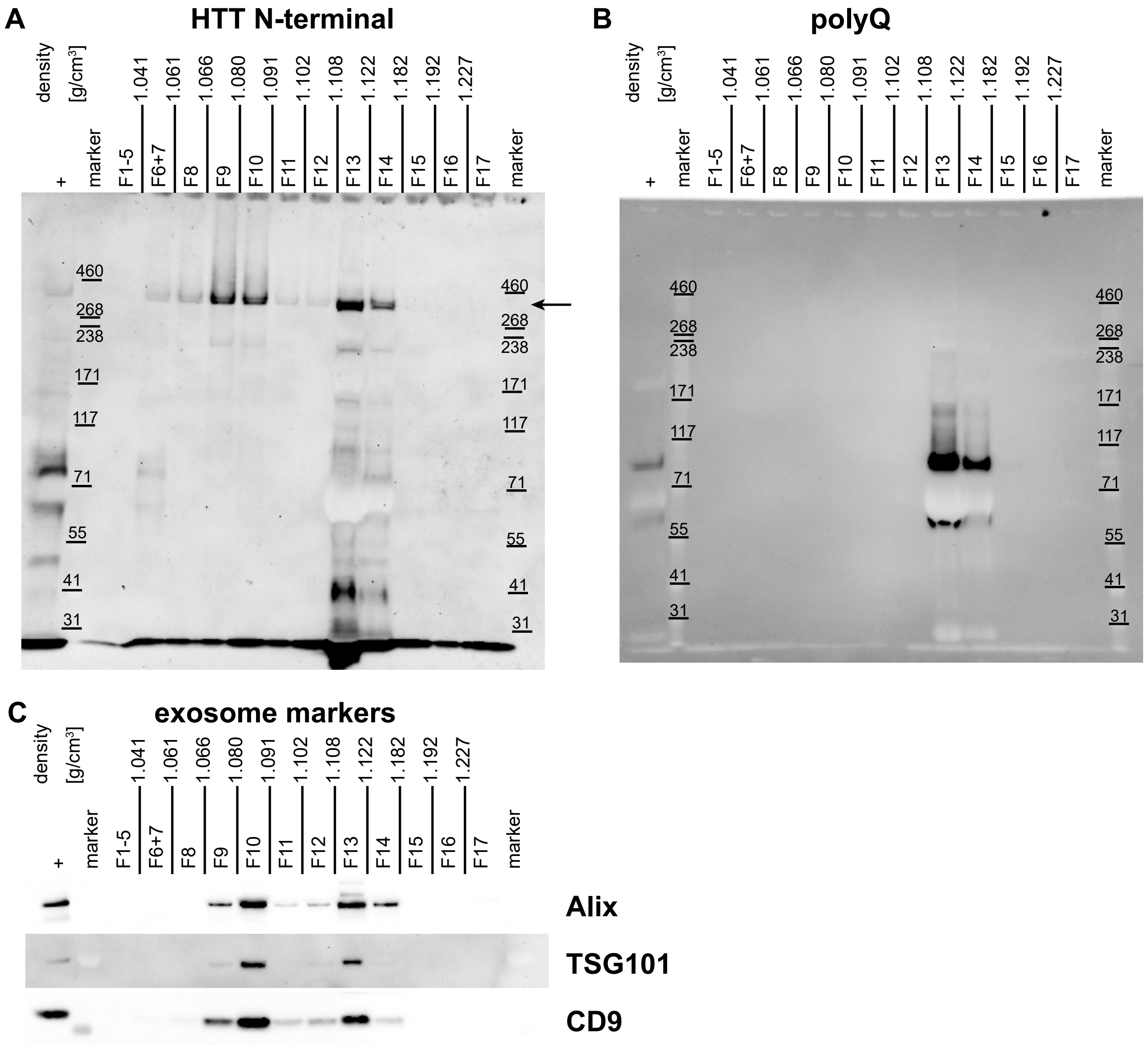

Figure S11

Huntingtin and exosome markers in EVs separated by density gradient ultracentrifugation in 18-months-old KI-HD pig. The 100,000 g pellet from the A31 knock-in pig plasma (sample labeled +) was overlaid by 40% to 5% Optiprep and separated by gradient ultracentrifugation. Seventeen fractions (1 ml each) were collected from top of the tube. The collected fractions were subjected to SDS-PAGE and western blotting to detect total huntingtin **(A)**, mutant huntingtin **(B)** and exosome markers Alix, TSG101 and CD9 **(C)**. Huntingtin appeared in fraction 13, which has the density 1.108-1.122 correspoding to exosomes and was also positive to exosome markers. Arrow indicates ~360 kDa full length huntingtin as a double band (the upper band corresponds to the mutant huntingtin with 85Q).

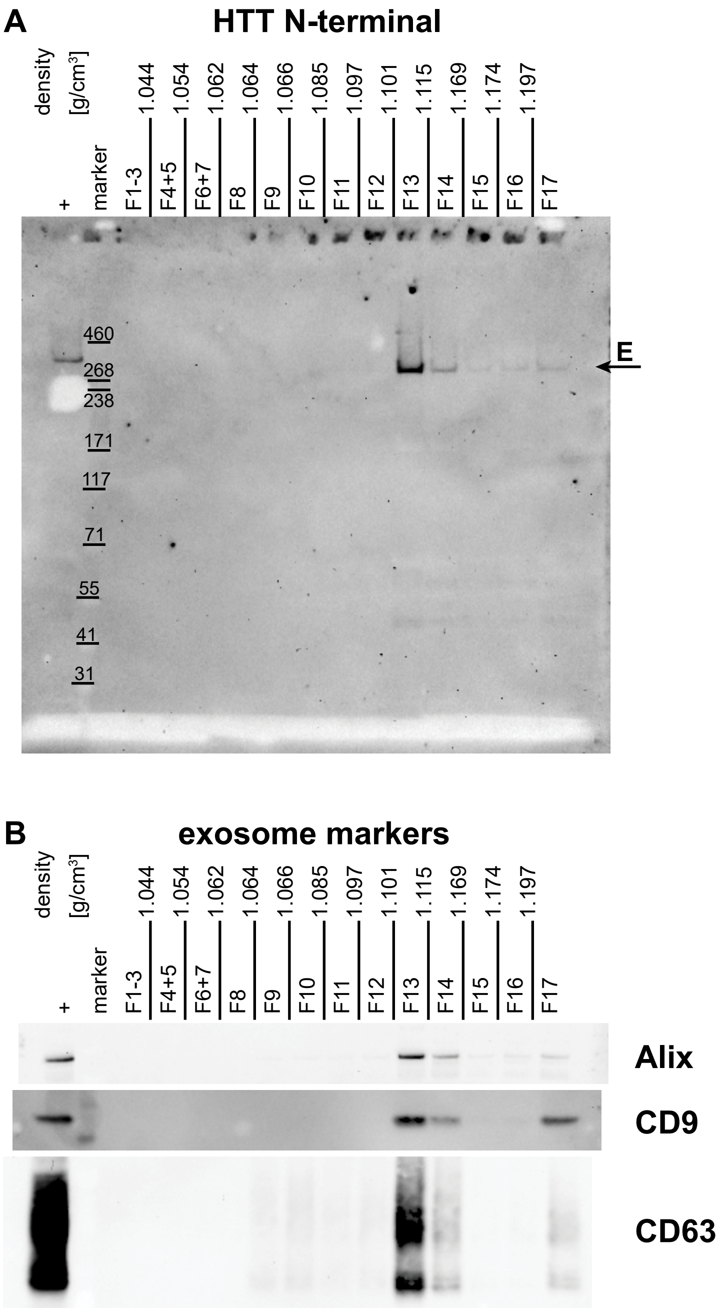

Figure S12

Huntingtin and exosome markers in EVs separated by density gradient ultracentrifugation of control human plasma. The 100,000 g pellet from the control human plasma (sample labeled +) was overlaid by 40% to 5% Optiprep and separated by gradient ultracentrifugation. Seventeen fractions (1 ml each) were collected from top of the tube. The collected fractions were subjected to SDS-PAGE and western blotting to detect total huntingtin **(A)**, and exosome markers Alix, CD9 and CD63 **(B)**. Huntingtin appeared in fractions 13 and 14, which had the density 1.101-1.169 correspoding to exosomes and were also positive to exosome markers. Arrow indicates ~360 kDa endogenous huntingtin protein.

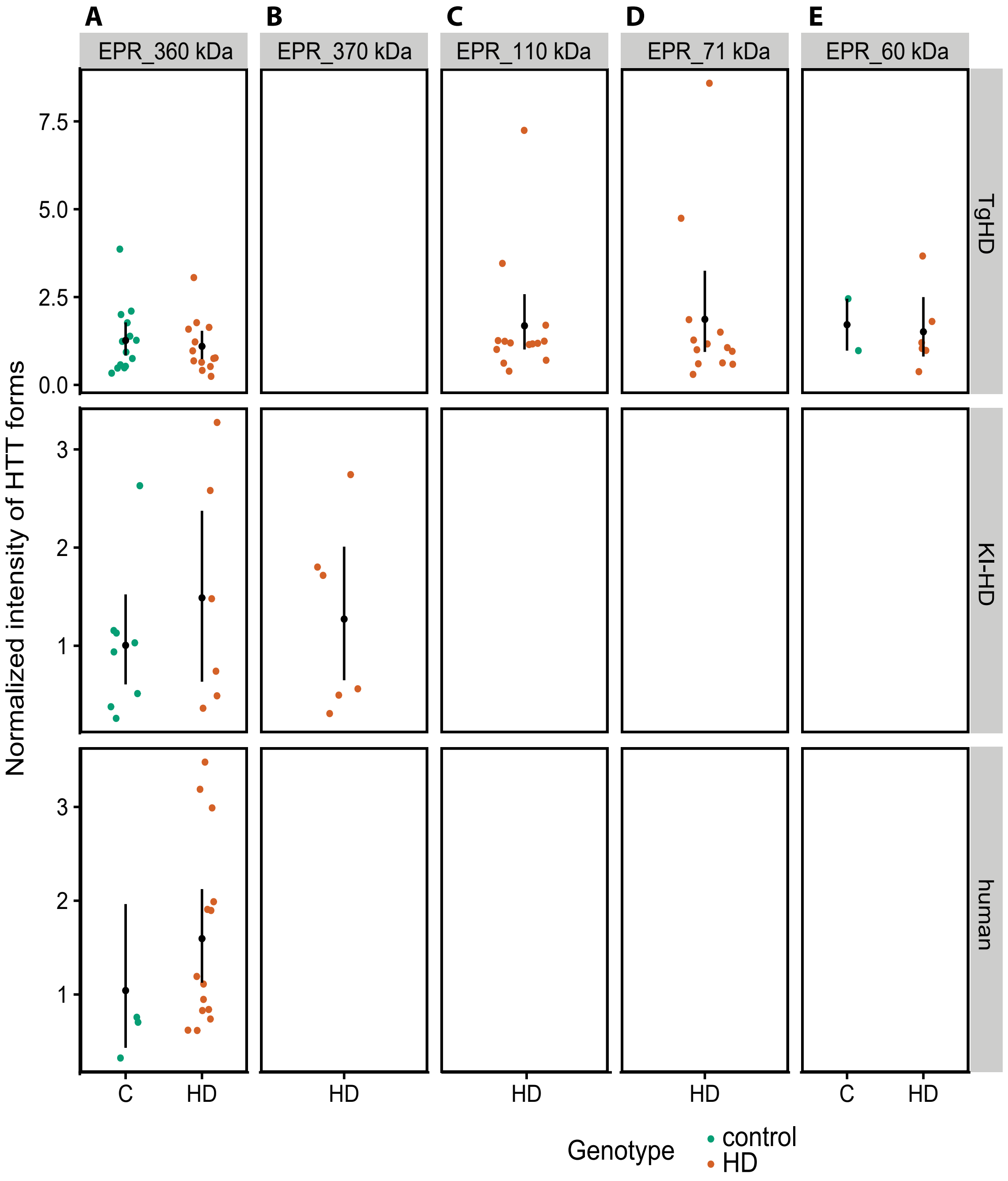

Figure S13

Normalized intensities of individual huntingtin forms in EVs from plasma of TgHD and KI-HD models and human plasma.

The HTT forms were detected by western blot using EPR5526 antibody recognizing N-terminal HTT/mHTT. The HTT band intensities were quantified by ImageLab software and normalized to CD9 intensities. **(A)** The full length HTT (~360 kDa) was detected in EVs of all models and in both HD and control samples. **(B)** For the KI-HD animals, the ~370 kDa band was typical, corresponding to full length HTT with elongated polyQ (85Q). **(C)** The ~110 kDa band is specific for TgHD animals and represents transgene translated into N-terminal 548 aminoacids of the human mHTT protein with 145Q. **(D)** The ~70 kDa mHTT fragment is produced in TgHD animals and is absent in WT controls. **(E)** In several control and TgHD animals, a 60 kDa HTT fragment was present, regardless on genotype.

Main findings

Transgenic pig model (TgHD)

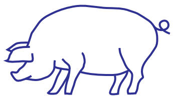

- HTT co-isolates with small EVs from blood plasma
- transgene (~110 kDa mHTT fragment) is present in EVs derived from plasma of TgHD pigs
- the transgene undergoes fragmentation and resulting ~70 kDa mHTT N-terminal fragment is specific for TgHD pig EVs
- significantly higher total HTT amount in TgHD EVs compared to wild type

Knock-in pig model (KI-HD)

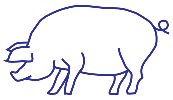

- HTT co-isolates with small EVs from blood plasma
- mHTT is present in EVs as full length (~370 kDa) protein, fragments are undetectable by used techniques
- higher, but not significantly, total HTT amouns in KI-HD pig EVs compared to wild type

HD patients

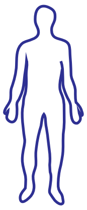

- HTT co-isolates with small EVs from blood plasma
- mHTT is weakly detectable as a full length protein, fragments are undetectable by used techniques
- total HTT levels in EVs of HD patients appear higher than in controls, but the difference is not significant

Further studies are needed using more sensitive techniques and high numbers of samples to verify if the HTT levels in blood-plasma derived EVs reflect the HD development.

Figure S14
Overview of main findings of the study.
